# Positive parenting moderates associations between childhood stress and corticolimbic structure

**DOI:** 10.1093/pnasnexus/pgad145

**Published:** 2023-06-13

**Authors:** Isabella Kahhalé, Kelly R Barry, Jamie L Hanson

**Affiliations:** Learning, Research, and Development Center, University of Pittsburgh, Pittsburgh, PA 15260, USA; Department of Psychology, University of Pittsburgh, Pittsburgh, PA 15260, USA; Department of Psychology, University of Houston, Houston, TX 77204, USA; Learning, Research, and Development Center, University of Pittsburgh, Pittsburgh, PA 15260, USA; Department of Psychology, University of Pittsburgh, Pittsburgh, PA 15260, USA

**Keywords:** adversity, early life stress, positive parenting, corticolimbic structure

## Abstract

Childhood stress has a deleterious impact on youth behavior and brain development. Resilience factors such as positive parenting (e.g. expressions of warmth and support) may buffer youth against the negative impacts of stress. We sought to determine whether positive parenting buffers against the negative impact of childhood stress on youth behavior and brain structure and to investigate differences between youth-reported parenting and caregiver-reported parenting. Cross-sectional behavioral and neuroimaging data were analyzed from 482 youth (39% female and 61% male, ages 10–17) who participated in an ongoing research initiative, the Healthy Brain Network (HBN). Regression models found that youth-reported positive parenting buffered against the association between childhood stress and youth behavioral problems (*β* = −0.10, *P* = 0.04) such that increased childhood stress was associated with increased youth behavior problems only for youth who did not experience high levels of positive parenting. We also found that youth-reported positive parenting buffered against the association between childhood stress and decreased hippocampal volumes (*β* = 0.07, *P* = 0.02) such that youth who experienced high levels of childhood stress and who reported increased levels of positive parenting did not exhibit smaller hippocampal volumes. Our work identifies positive parenting as a resilience factor buffering youth against the deleterious impact of stressful childhood experiences on problem behaviors and brain development. These findings underscore the importance of centering youth perspectives of stress and parenting practices to better understand neurobiology, mechanisms of resilience, and psychological well-being.

**Significance Statement**

Childhood stress (e.g. the death of a loved one or illness) can have a negative impact on mental health and well-being; it is therefore critical to investigate resilience factors that might protect against stress. We consider positive (i.e. warm and supportive) parenting as a buffer against the impacts of childhood stress on youth behavior problems and corticolimbic structure. Youth perceptions of positive parenting moderated the association between childhood stress and (i) youth behavioral problems and (ii) hippocampal volumes. No significant associations emerged from interactions with caregiver-reported positive parenting or amygdala volumes. We conclude that positive parenting may function as a key resilience factor protecting youth from the deleterious impact of stressful experiences and that youth perspectives are critical to understanding how life stress shapes neurodevelopment and behavior.

## Introduction

Stress in childhood and adolescence can significantly compromise mental health and well-being (1). A growing body of work has noted that childhood stress is related to alterations in key neural components of the stress response and changes in brain development, including in corticolimbic brain areas such as the amygdala and the hippocampus (2, 3). The amygdala, a central neural hub for vigilance and processing negative emotions (4), and the hippocampus, an area involved in memory representations and servicing goal–directed behavior (5), are critical components of the limbic system's regulation of emotion processing and behavioral responding. Smaller hippocampal volumes have consistently been found in samples exposed to various forms of stress including physical abuse and neglect (6, 7). Patterns of structural differences in the amygdala are less uniform, with work in pediatric populations exposed to stress finding larger volumes (8), smaller volumes (9), and no differences (10, 11). These diverging results may be due to nonlinear impacts of stress on amygdala neurobiology across the lifespan (2).

Resilience factors such as relationships with caregivers can significantly protect against the many deleterious developmental outcomes associated with childhood stress, with more positive caregiver–child connections being associated with better adjustment and fewer problem behaviors among youth (12, 13). Variations in parenting are also related to functional and structural brain differences among children (14). Longitudinal studies have found that positive parenting behaviors, such as being warm, validating, and responsive, relate to larger hippocampal volumes (15) and attenuated growth of amygdala and prefrontal cortex volumes (16). These neurobiological associations may cascade to behavioral variations in cognitive control, emotion, and reward-related behaviors, though evidence in this space is limited.

While normative variations in parenting shape brain development, it remains less clear whether positive parenting can buffer against the negative impacts of stress on neurobiology. Several studies have considered interactions between positive parenting and childhood stress on the amygdala and hippocampus. Results from these studies have been inconsistent and have not clarified the potential buffering effect of positive parenting. Some results suggest *slower* amygdala growth over time for youth experiencing high levels of positive parenting and high stress, *accelerated* amygdala growth for youth experiencing high levels of positive parenting and less stress, and no significant effects of stress and parenting on hippocampal volumes (17). In contrast, others have found *larger* hippocampal and amygdala volumes in adolescents who had low preschool stress and high maternal support (18). These investigators noted that caregiver support did not actually buffer against stress, as high positive parenting was strongly associated with the development of these regions only in the context of low stress exposure. Finally, work from an adult sample found that increased poverty-related stress was associated with smaller amygdala and hippocampal volumes, but not for those who had completed an intervention focused on boosting youth self-regulation and positive parenting practices in caregivers (19). It is unclear across these studies whether interactions between stress and parenting are associated with both the hippocampus and amygdala, and whether there is a buffering role of parenting against any effect of stress on brain volumes.

Past studies have employed modest sample sizes (*N* < 150) and typically focus on stressful contexts (i.e. socioeconomic disadvantage) and not the actual experience of stress (17–19). There is a need to consider a variety of stressful life events in one's childhood above and beyond associations with socioeconomic status, particularly in light of the stressors posed by the COVID-19 pandemic and connected declines in youth mental health (20). Additionally, emerging work suggests that self-perceptions of experiences (rather than objective measures) are powerful predictors of outcomes. For example, childhood maltreatment was related to elevated rates of adult psychopathology only when considering self-report experiences of maltreatment and not when relying on court-documented records alone (21); a similar pattern has been replicated for other early life experiences such as peer victimization and neighborhood violence (22, 23). Focusing on youth perceptions during late childhood to adolescence may be particularly critical given that this period is marked by significant social, cognitive, and affective changes. Further, research has highlighted common correlates of altered corticolimbic structure; for example, there are connections between smaller hippocampal volumes and psychopathology (24), which suggests the importance of modeling confounds when examining associations between childhood stress and corticolimbic structure.

Motivated by gaps present in this nascent body of work, we considered relations between childhood stress, parenting, and corticolimbic structures in a large neuroimaging sample, specifically centering youth perspectives. We first examined if stress and parenting were related to youth behavioral problems, expecting that positive parenting would moderate an association between childhood stress and behavior. We next examined if stress was related to volumetric differences in the amygdala and hippocampus and if parenting moderated these relations. We hypothesized that greater positive parenting would attenuate the negative association between stress and hippocampal volumes. Given inconsistencies in prior work examining stress amygdala volumes, we did not predict main or interactive effects of stress and parenting on the amygdala (i.e. analyses were exploratory in nature). We were particularly interested in investigating differences between youth versus caregiver perceptions of positive parenting, predicting stronger interactive effects for youth reports of positive parenting compared with caregiver reports.

## Method

### Participants

Data from 482 participants (39% female and 61% male) between the ages of 10–17 years with T1-weighted structural images were analyzed from the ongoing Healthy Brain Network (HBN) research initiative (see sample characteristics in Table 1). For additional information about the HBN sample, please see the HBN data descriptor (25) and our Supplementary material (see Table S2 for sample characteristics per study recruitment site and Table S4 for a correlation table of key study variables). All questionnaires showed “good” to “excellent” internal consistency (Cronbach's *α* > 0.7, as detailed in our Supplementary material).

**Table 1.** Descriptive statistics for key variables.

Characteristic	Value^a^
Total	*N* = 482
Age	13.34 (2.42)
Sex	
Female	190 (39%)
Male	292 (61%)
Income	
Less than $10,000	10 (2.4%)
$10,000 to $19,999	11 (2.6%)
$20,000 to $29,999	12 (2.9%)
$30,000 to $39,999	16 (3.8%)
$40,000 to $49,999	18 (4.3%)
$50,000 to $59,999	11 (2.6%)
$60,000 to $69,999	16 (3.8%)
$70,000 to $79,999	12 (2.9%)
$80,000 to $89,999	26 (6.2%)
$90,000 to $99,999	20 (4.8%)
$100,000 to $149,999	81 (19%)
$150,000 or more	128 (31%)
Choose not to disclose	55 (13%)
Positive parenting: youth	21 (5)
Positive parenting: caregiver	25 (3)
Negative life events: youth	2.84 (1.17)
Negative life events: caregiver	2.52 (1.01)
Behavioral problems: youth	48 (26)
Behavioral problems: caregiver	11 (6)
Total gray matter volume^b^	745 (76)^b^
Imaging site	
CBIC	186 (39%)
CUNY	14 (2.9%)
RU	200 (41%)
SI	82 (17%)

a *N* = *N*; mean (SD); *n* (%).

b Total gray matter volume values are divided by 1,000.

CBIC, Citigroup Biomedical Imaging Center; CUNY, The City University of New York; RU, Rutgers University; SI, Staten Island. Units of measurement are the following: age (years); positive parenting: youth/caregiver (raw scores from the Alabama Parenting Questionnaire: positive parenting subscale); negative life events: youth/caregiver (Negative Life Events Scale, average upsetness score); behavioral problems: youth (Youth Self Report Total Behavioral Problems Raw Score); behavioral problems: caregiver (Strength and Difficulties Questionnaire Total Raw Score); and total gray matter volume (voxels divided by 1,000).

### Childhood stress

Childhood stress was operationalized through the Negative Life Events Scale (NLES), a checklist measuring family-, community-, and school-based stressors (26). Due to our focus on youth perceptions of their experiences, we used average youth-rated upsetness as our measure of childhood stress. Children and caregivers rated how upset they were in response to stressful events they had experienced (0 = not at all upset to 4 = very upset), with a higher score reflecting increased distress in response to negative events.

### Positive parenting

Youth report and caregiver report of positive parenting were measured using the positive parenting subscale from the Alabama Parenting Questionnaire (APQ) (27). For each item, youth and caregivers rated how often (1 = never to 5 = always) they typically experienced (or performed) a specific kind of parenting behavior.

### Youth behavioral functioning

Youth behavioral functioning was operationalized by the Youth Self-Report (YSR) Total Raw Score. The YSR is a well-validated and widely used 112-item instrument for problem behaviors across the internalizing and externalizing spectrums among children aged 11–18 (28).

### MRI data acquisition and processing

High-resolution T1-weighted anatomical MRI scans were collected from four sites in the New York City area. We performed standard-processing approaches from FreeSurfer (version 7.1) and excluded participants with low-quality images and high-motion scans (see supplementary material and Table S1 for details on MRI data, acquisition, processing, and quality control).

### Statistical analysis

All R codes can be found on our study GitHub (https://github.com/isabellakahhale/CorticoLimbicParenting). Regression models examined the interactions of stress and parenting in predicting youth behavioral problems and included sex, age, and scanning site as covariates. We fit linear mixed-effects models (LMEMs) for each brain region of interest (i.e. total hippocampal volume and total amygdala volume) due to potential variations in research sites and scanners. LMEMs included a random effect of the imaging site and sex, age, and estimated total intracranial volume as covariates. We ran separate models testing interactions between negative life events and a) youth-reported positive parenting and b) caregiver-reported positive parenting for each brain region of interest. For significant interaction terms, we conducted simple slope analyses (i.e. compared the mean of all individuals, +1 SD, and −1 SD of the mean). Across models, we tested for differences between any significant and nonsignificant interaction terms (see Supplementary material for details). Our Supplementary material contains regression diagnostics and sensitivity/supplemental analyses to probe the robustness of our effects. These include the following: (i) examinations of participant age distributions by sex (Fig. S1), (ii) analysis of both caregiver and youth reports of our major variables (i.e. positive parenting, negative life events, and youth behavioral problems) within our main models, (iii) main effect models without interaction terms, (iv) use of general additive mixed models (GAMMs) to account for nonlinear age effects, (v) modeling stress with a nonlinear quadratic term, (vi) covarying total gray matter volume as an alternative brain scaling variable, (vii) considering left and right hemispheres of brain regions separately, (viii) examining associations between hippocampal volumes and youth behavioral problems, and (ix) comparing key variables between the HBN imaging sample to the total HBN sample. Additionally, the presence of psychopathology may be a potential confound in the association between childhood stress and altered corticolimbic structure, given found links between smaller hippocampal volumes and both childhood stress (29) and psychopathology (24). We ran analyses considering a binary variable indicating the presence of any psychopathology diagnoses in a subsample of the available data (*N* = 226) (see Table S3 for detailed psychopathology data). We also considered the effect of four operationalizations of socioeconomic status (SES) given prior work connecting socioeconomic disadvantage to altered corticolimbic structure and our interest in testing the unique contribution of actual experience of stress on youth behavioral problems, corticolimbic structure, and the moderating effect of positive parenting. We ran statistical corrections for multiple comparisons on our main models, and when reporting significant statistics, we provide both raw *P*-values and Storey-corrected *P*-values (*q*-values derived using a false discovery rate [FDR]-corrected approach) (30).

## Results

Descriptive statistics are found in Table 1 and in our Supplementary material.

### Stress on behavior

**Fig. 1.**
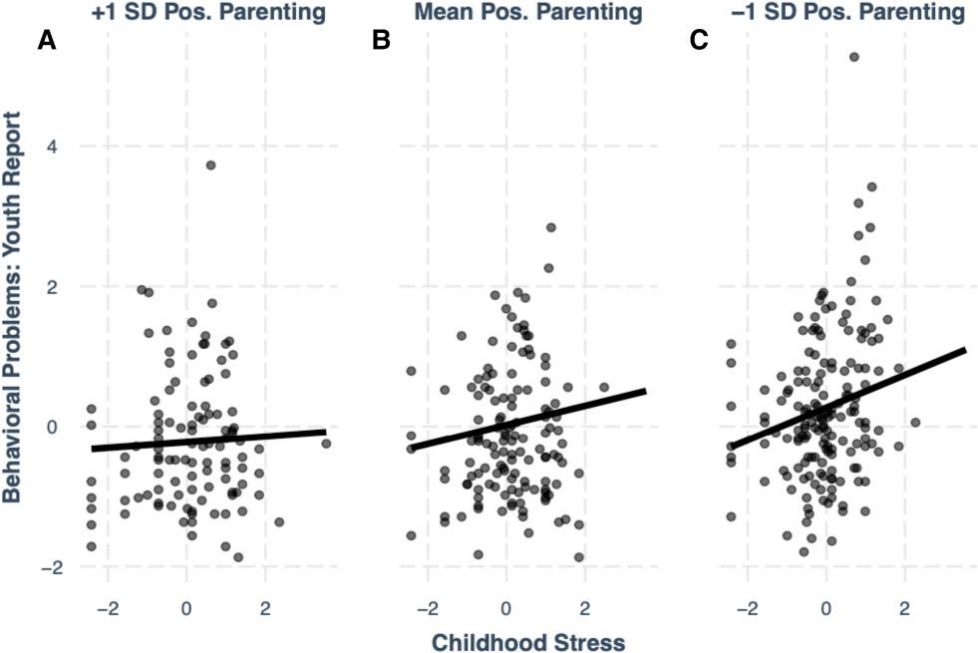
The association between childhood stress and youth behavioral problems at three levels of positive parenting. Youth reporting higher (+1 SD of positive parenting) are shown in A), youth with mean levels of positive parenting are shown in B), and youth with lower levels of positive parenting (−1 SD) are shown in C). Stress is on the horizontal axis and behavioral problems (scaled, not raw YSR values) are on the vertical axis.

We first considered the moderating effect of youth-reported positive parenting on the association between negative life events and youth-reported behavioral problems (Fig. 1). Results showed a significant main effect of negative life events (*β* = 0.14, *P*_raw_ < 0.001, *P*_corrected_ = 0.017) and a significant main effect of positive parenting (*β* = −0.25, *P*_raw_ < 0.001, *P*_corrected_ < 0.001). We also found a significant interaction between negative life events and positive parenting (*β* = −0.10, *P*_raw_ = 0.040, *P*_corrected_ = 0.052). Simple slope analyses indicated that the association between negative life events and behavioral problems was significant at lower (*β* = 0.24, *P*_raw_ < 0.001, *P*_corrected_ = 0.049) and average (*β* = 0.14, *P*_raw_ = 0.005, *P*_corrected_ = 0.016) levels of positive parenting, but not at high levels of positive parenting. There was no relation between stress and behavioral problems when youth were exposed to the highest levels of positive parenting, suggesting a buffering effect. We considered a similar model examining the interaction between stress and positive parenting on behavioral problems using caregivers as the informant of positive parenting. We found no buffering effect of positive parenting against the association between stress and behavioral problems using the caregiver report measure of positive parenting. Models without interaction terms and caregiver report measures of behavioral problems and negative life stress can be found in the Supplementary material.

### Stress and positive parenting on hippocampal volume

**Fig. 2.**
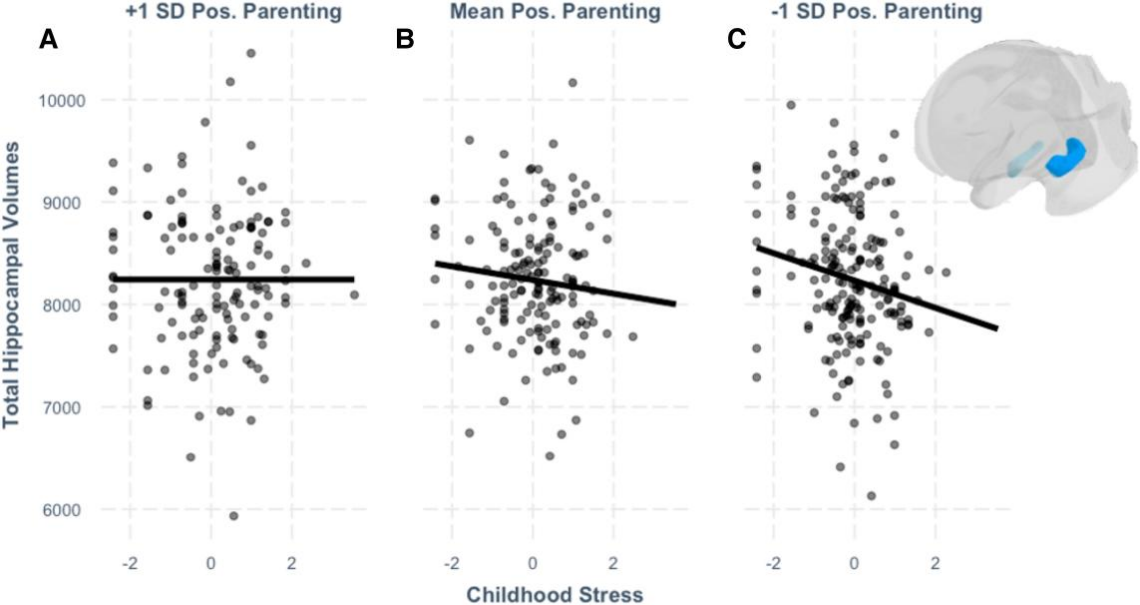
The association between childhood stress and total hippocampal volume at three levels of positive parenting. Youth reporting higher (+1 SD of positive parenting) are shown in A), youth with mean levels of positive parenting are shown in B), and youth with lower levels of positive parenting (−1 SD) are shown in C). Stress is on the horizontal axis and hippocampal volume is on the vertical axis. In the right corner is a 3D figure showing the hippocampus, our region of interest. The hippocampus is depicted in blue and is shown on a transparent rendering of the whole brain.

We next considered the moderating effect of youth-reported positive parenting on the association between negative life events in childhood and hippocampal volumes (see Table 2 for model output). Results from this model showed a significant negative main effect of stress (*β* = −0.07, *P*_raw_ = 0.029, *P*_corrected_ = 0.042) on the total hippocampal volume. As predicted, there was a significant interaction between youth-reported positive parenting and stress (*β* = 0.07, *P*_raw_ = 0.033, *P*_corrected_ = 0.045, Fig. 2). Simple slope analyses indicated that, for those with average or below-average levels of positive parenting, increased reports of negative life events were associated with decreased hippocampal volumes (average positive parenting *β* = −0.07, *P*_raw_ = 0.028, *P*_corrected_ = 0.042; below-average positive parenting *β* = −0.14, *P*_raw_ = 0.005, *P*_corrected_ = 0.016). There was no effect of youth-reported negative life events on hippocampal volumes for participants reporting above-average positive parenting. This finding underscores the stress-buffering role of positive parenting given that reports of negative life events had a negative association with hippocampal volumes only in the presence of below-average and average levels of positive parenting, and not in the presence of above-average positive parenting. Sensitivity analyses testing left and right hippocampal volumes individually can be found in the Supplementary material, with results consistent with the patterns for total hippocampal volume.

**Table 2.** LMER output comparing child report and caregiver report models for hippocampal volumes.

Predictors	Total hippocampal volume: child report model			Total hippocampal volume: caregiver report model		
	Estimates	CI	*P*	Estimates	CI	*P*
Intercept	−0.14	−0.51 to 0.22	0.449	−0.14	−0.51 to 0.23	0.465
Scan quality	0.09	0.01–0.16	**0.023**	0.09	0.01–0.16	**0.027**
Sex	0.12	−0.03 to 0.27	0.116	0.12	−0.03 to 0.28	0.107
Age	0.07	0.00–0.13	**0.044**	0.07	0.01–0.14	**0.023**
Socioeconomic status	0.06	−0.0 to 0.12	0.100	0.06	−0.00 to 0.13	0.059
Total intracranial volume	0.59	0.52–0.66	**<0.001**	0.59	0.51–0.66	**<0.001**
Negative life events	−0.07	−0.13 to −0.01	**0.029**	−0.06	−0.12 to 0.00	0.062
Positive parenting youth report	0.01	−0.06 to 0.07	0.879			
Negative life events × positive parenting youth report	0.07	0.01–0.13	**0.033**			
Positive parenting caregiver report				0.04	−0.03 to 0.10	0.247
Negative life events × positive parenting caregiver report				0.01	−0.05 to 0.07	0.730
**Random effects**						
*σ*^2^	0.47			0.47		
*τ*_00_	0.12_Site_			0.13_Site_		
ICC	0.21			0.21		
*N*	4_Site_			4_Site_		
Observations	475			474		
Marginal *R*^2^/conditional *R*^2^	0.421/0.540			0.415/0.539		

The left model output considers an interaction between youth-reported positive parenting and childhood stress on total hippocampal volumes. The right model output considers an interaction between caregiver-reported positive parenting and childhood stress on the total hippocampal volumes. Both models included the same covariates (scan quality, sex, age, and estimated total intracranial volume) and a random effect of the imaging site. Bolded values indicate effects that were significant at *P* < .05.

Next, we considered caregiver-reported positive parenting and did not find a significant interaction between negative life events and caregiver-reported positive parenting (*β* = 0.02, *P*_raw_ = 0.730) on total hippocampal volume. We tested differences between interaction terms for our two models (i.e. one model considering youth-reported positive parenting and one model considering caregiver-reported positive parenting, see Table 2). Our results indicate that the interaction terms from the youth-reported and caregiver-reported models were significantly different (*t* = 2.31, *P*_raw_ < 0.021) (see Supplementary material for details).

### Stress and positive parenting on amygdala volume

There was not a significant main effect of negative life events (*β* = −0.04, *P*_raw_ = 0.303) on total amygdala volume. There were also no interactive effects between negative life events and youth-reported positive parenting on total amygdala volume (*β* = 0.02, *P*_raw_ = 0.563; Table S3). There were no interactive effects between negative life events and caregiver-reported positive parenting on the total amygdala volume (*β* = −0.02, *P*_raw_ = 0.587).

### Sensitivity and supplemental analyses

We constructed additional models to determine the robustness of our effects (see Supplementary material). These analyses indicate that (i) interactions between negative life events and youth-reported positive parenting are associated with both youth- and caregiver-reported behavioral problems, (ii) interactions between negative life events and youth-reported positive parenting on hippocampal volumes remain consistent when considering left and right hippocampal volumes, using different covariates in the model, or using different model specifications (i.e. generalized additive mixed models) to model potential nonlinear impacts of age, and (iii) negative life events and caregiver-reported positive parenting do not interact to predict either youth-reported behavioral problems, caregiver-reported behavioral problems, or hippocampal volume. We also report a significant association between left hippocampal volumes and caregiver-reported youth behavioral functioning. Supplemental analyses explored potentially confounding associations between effects of interest and psychopathology given the well-replicated links between psychopathology, childhood stress, and hippocampal volumes. Of note, the sample size for our analysis involving the psychopathology variable was drastically reduced due to missing data (*N* = 226). In this subsample, adding the presence of a mental health diagnosis was not significantly associated with the total hippocampal volume.

We ran additional analyses examining associations with several markers of socioeconomic status given connections between socioeconomic status and hippocampal volumes. Results indicated that the potentially comorbid and stressful experience of the socioeconomic environment did not account for observed effects of negative life events and positive parenting on youth behavioral problems and hippocampal volumes (see Supplementary material). To account for the comorbid influence of socioeconomic status, a variable operationalizing socioeconomic status (via the Barratt Simplified Measure of Social Status parental education and occupational “prestige”) was added as a covariate in all main analyses.

## Discussion

We found three major results: (i) negative life events and youth perceptions of positive parenting are associated with behavioral problems, (ii) negative life events are significantly associated with smaller hippocampal volumes, and (iii) positive parenting moderates this association such that youth who reported high levels of positive parenting did not show smaller hippocampal volumes even with increasing levels of stress. Only youth perspectives of positive parenting interacted with childhood stress (measured through negative life events) to predict hippocampal volumes; caregiver report of positive parenting was not related to neurobiology as either a main effect or in an interaction with stress. Such findings underscore the importance of including youth as reporters of their own experiences to better understand consequences for neurodevelopment and behavior (31). We did not find significant associations with childhood stress, parenting, and amygdala volumes.

Our results underscore well-replicated connections between exposure to childhood stress and smaller volumes in the hippocampus (29, 32). There were no significant associations between amygdala volumes and childhood stress. This is consistent with theoretical work suggesting nonlinear alterations in the amygdala structure related to stress (2) and empirical studies connecting stress with both enlarged (8) and shrunken (9) amygdala (versus clear connections between stress and smaller hippocampal volumes). This directionality may depend more on the trajectory of adversity across development and therefore be more appropriately modeled by longitudinal research designs.

Positive parenting can provide youth with a stable environment to learn social skills, feel supported, and practice cognitive and behavioral regulation. Other work examining interactive effects between childhood stress and parenting on brain volumes has not found evidence of a buffering effect of parenting, instead suggesting that warm and supportive parenting was a compounding positive force in the context of low stress (18). In contrast, we report interactions highlighting the stress-buffering effect of parenting on neurobiology. This aligns with the well-replicated body of work establishing that high-quality parenting is associated with resilience in youth through adulthood (33). Across diverse samples of families, interventions that focus on increasing positive and supportive parenting are associated with better outcomes across socioemotional and cognitive domains in normative and adversity-exposed samples of youth (14, 34).

The protective effect of parenting against the deleterious neurobiological impacts of stress may function through modulating biological and socioemotional processes such as hypothalamic–pituitary–adrenal (HPA) axis responsiveness, cortisol reactivity, and self-regulation. Longitudinal work has found that parenting influences HPA axis and cortisol reactivity, which in turn impacts hippocampal volume during development (35). In addition to HPA axis changes, parenting is likely to influence other skills that may cascade to impact neurobiology (36). For example, we have noted alterations in hippocampal neurobiology after interventions boosting self-regulation (37).

Previous studies have relied on adult informants of caregiving practices, childhood stress, and youth behavioral outcomes; however, youth are active agents within their developmental context and are attuned to environmental stressors (38). The modest correlation (*r* = 0.22) between youth and caregiver reports of positive parenting in this sample aligns with previous work establishing small to moderate correlations between youth and caregiver reports of behavioral and emotional functioning (39). Additionally, youth reports of stress are often more directly associated with increased risks of psychopathology and other negative developmental outcomes compared with reports from adult informants (38, 40). The current study fills the gap in the literature by centralizing youth perspectives on parenting, stress, and behavior in order to understand how youths’ own experiences and emotions ultimately shape neurodevelopmental trajectories and psychological functioning (41).

Sensitivity analyses to determine the specificity of our effects considered the confounding roles of psychopathology and the potentially stressful experience of socioeconomic status. We found that the presence of a mental health diagnosis was not significantly associated with total hippocampal volumes in a subset of our sample (*N* = 266), suggesting that the observed interaction between childhood stress and youth-reported positive parenting on hippocampal volumes in our larger sample is not explained by comorbid psychopathology. Similarly, we considered associations with various operationalizations of socioeconomic status considering that socioeconomic disadvantage can be an experience of stress that has been associated with altered corticolimbic structure (17, 19). Notably, even after controlling for potential confounds (e.g. caregiver's level of education and occupational prestige), the interaction between childhood stress and youth-reported positive parenting on youth behavioral problems and hippocampal volumes remained significant.

We note several limitations that could serve as important directions for future research. First, we report significant associations that are small in magnitude, with effect size estimates indicating that childhood stress at below-average parenting explains 0.06% of the variance in youth behavioral problems; similarly, childhood stress at below-average parenting explains 0.02% of the variance in total hippocampal volumes. Youth behavioral problems and total hippocampal volumes were also not significantly associated in our supplemental analyses. Of note, we did find a significant association between youth behavioral problems and left hippocampal volumes (detailed in our Supplementary material). Second, our cross-sectional design limits the ability to investigate causal impacts of stress and parenting. Third, our sample included a wide age range spanning childhood to adolescence. Future work would benefit from longitudinal methods and targeted age ranges to parse effects of stress and parenting across development, especially given that there may be critical periods related to the buffering effects of parenting (42). Assessing pubertal status, timing, and pubertal hormones may provide one such avenue to examine differential developmental sensitivity to parenting and stress-related factors. Fourth, we used an aggregated measure of stress to examine the impact of subjective distress of a variety of potential negative events, precluding a fine-tuned assessment of which stressors and the duration of exposure impact behavior and neurobiology (43). Fifth, only one domain of parenting was examined to understand how positive experiences shape trajectories of hippocampal development. Concentrating on positive parenting allowed us to examine links with resilience and strength-based processes that may shape hippocampal structure; at the same time, previous literature emphasizes that negative aspects of parenting (e.g. harshness and psychological control) can increase psychopathology risk and decrease well-being (44). Finally, there may be sex-specific effects for neurobiology related to stress, parenting, and their interaction, as there is at least one report of sex differences related to stress, parenting, and neurobiology (17). However, three-way interactions require extremely large samples (*N* > 1,300) for consistency in findings (45) and appropriate levels of Type I error (46).

Our findings have implications for understanding the impact of childhood stress as well as the factors that buffer youth against the noxious consequences of stress. Given the critical role of the hippocampus in memory and goal-directed behavior, alterations in this structure may create vulnerabilities for later negative outcomes. While additional research is needed to clarify complex relations between stress, parenting, and the brain, our data provide insight into how environmental forces may interact to influence neurobiology.

## Supplementary Material

pgad145_Supplementary_Data

## Data Availability

Behavioral and neuroimaging data used in our analyses were sourced from the Healthy Brain Network (25). These data are publicly and freely available from the Child Mind Institute (http://fcon_1000.projects.nitrc.org/indi/cmi_healthy_brain_network/). Data and documentation are provided online at this website.

## References

[1] Cohen S , Murphy ML, Prather AA. 2019. Ten surprising facts about stressful life events and disease risk. Annu Rev Psychol. 70:577–597. 29949726 10.1146/annurev-psych-010418-102857 PMC6996482

[2] Hanson JL , Nacewicz BM. 2021. Amygdala allostasis and early life adversity: considering excitotoxicity and inescapability in the sequelae of stress. Front Hum Neurosci. 15:624705. 10.3389/fnhum.2021.624705 PMC8203824 34140882

[3] McEwen BS , Nasca C, Gray JD. 2016. Stress effects on neuronal structure: hippocampus, amygdala, and prefrontal cortex. Neuropsychopharmacol. 41(1):3–23. 10.1038/npp.2015.171 PMC4677120 26076834

[4] Adolphs R . 2010. What does the amygdala contribute to social cognition? Ann N Y Acad Sci. 1191(1):42–61. 20392275 10.1111/j.1749-6632.2010.05445.x PMC2871162

[5] Murty VP , Calabro F, Luna B. 2016. The role of experience in adolescent cognitive development: integration of executive, memory, and mesolimbic systems. Neurosci Biobehav Rev. 70:46–58. 27477444 10.1016/j.neubiorev.2016.07.034 PMC5074888

[6] Hanson JL , Nacewicz BM, Sutterer MJ, et al 2015. Behavioral problems after early life stress: contributions of the hippocampus and amygdala. Biol Psychiatry. 77(4):314–323. 24993057 10.1016/j.biopsych.2014.04.020 PMC4241384

[7] McLaughlin KA , Weissman D, Bitrán D. 2019. Childhood adversity and neural development: a systematic review. Annu Rev Dev Psychol. 1:277–312. 32455344 10.1146/annurev-devpsych-121318-084950 PMC7243625

[8] Tottenham N , Hare TA, Quinn BT, et al 2010. Prolonged institutional rearing is associated with atypically large amygdala volume and difficulties in emotion regulation. Dev Sci. 13(1):46–61. 20121862 10.1111/j.1467-7687.2009.00852.x PMC2817950

[9] Saxbe D , Khoddam H, Piero LD, et al 2018. Community violence exposure in early adolescence: longitudinal associations with hippocampal and amygdala volume and resting state connectivity. Dev Sci. 21(6):e12686. 10.1111/desc.12686 PMC11694245 29890029

[10] Butler O , Yang XF, Laube C, Kühn S, Immordino-Yang MH. 2018. Community violence exposure correlates with smaller gray matter volume and lower IQ in urban adolescents. Hum Brain Mapp. 39(5):2088–2097. 29450935 10.1002/hbm.23988 PMC6866594

[11] Hodel AS , et al 2015. Duration of early adversity and structural brain development in post-institutionalized adolescents. NeuroImage 105:112–119. 25451478 10.1016/j.neuroimage.2014.10.020 PMC4262668

[12] Evans GW , Kim P, Ting AH, Tesher HB, Shannis D. 2007. Cumulative risk, maternal responsiveness, and allostatic load among young adolescents. Dev Psychol. 43(2):341–351. 17352543 10.1037/0012-1649.43.2.341

[13] Ge X , Natsuaki MN, Neiderhiser JM, Reiss D. 2009. The longitudinal effects of stressful life events on adolescent depression are buffered by parent–child closeness. Dev Psychopathol. 21(2):621–635. 19338701 10.1017/S0954579409000339

[14] Farber MJ , Gee DG, Hariri AR. 2020. Normative range parenting and the developing brain: a scoping review and recommendations for future research. Eur J Neurosci. 55:2341–2358. 33051903 10.1111/ejn.15003 PMC8044268

[15] Luby J , Belden A, Harms MP, Tillman R, Barch DM. 2016. Preschool is a sensitive period for the influence of maternal support on the trajectory of hippocampal development. Proc Natl Acad Sci USA. 113(20):5742–5747. 27114522 10.1073/pnas.1601443113 PMC4878487

[16] Whittle S , Simmons JG, Dennison M, et al 2014. Positive parenting predicts the development of adolescent brain structure: a longitudinal study. Dev Cogn Neurosci. 8:7–17. 24269113 10.1016/j.dcn.2013.10.006 PMC6990097

[17] Whittle S , Vijayakumar N, Simmons JG, et al 2017. Role of positive parenting in the association between neighborhood social disadvantage and brain development across adolescence. JAMA Psychiatry 74(8):824–832. 28636697 10.1001/jamapsychiatry.2017.1558 PMC5710640

[18] Luby J , Tillman R, Barch DM. 2019. Association of timing of adverse childhood experiences and caregiver support with regionally specific brain development in adolescents. JAMA Netw Open. 2(9):e1911426. 10.1001/jamanetworkopen.2019.11426 PMC6751767 31532514

[19] Brody GH , Gray JC, Yu T, et al 2017. Protective prevention effects on the association of poverty with brain development. JAMA Pediatr. 171(1):46–52. 27893880 10.1001/jamapediatrics.2016.2988 PMC5214580

[20] Magson NR , et al 2021. Risk and protective factors for prospective changes in adolescent mental health during the COVID-19 pandemic. J Youth Adolescence. 50(1):44–57. 10.1007/s10964-020-01332-9 PMC7590912 33108542

[21] Danese A , Widom CS. 2020. Objective and subjective experiences of child maltreatment and their relationships with psychopathology. Nat Hum Behav. 4(8):811–818. 32424258 10.1038/s41562-020-0880-3

[22] Goldman-Mellor S , Margerison-Zilko C, Allen K, Cerda M. 2016. Perceived and objectively-measured neighborhood violence and adolescent psychological distress. J Urban Health. 93(5):758–769. 27604615 10.1007/s11524-016-0079-0 PMC5052152

[23] Gromann P , Goossens F, Olthof T, Pronk J, Krabbendam L. 2013. Self-perception but not peer reputation of bullying victimization is associated with non-clinical psychotic experiences in adolescents. Psychol Med. 43(4):781–787. 22895003 10.1017/S003329171200178X

[24] Videbech P , Ravnkilde B. 2004. Hippocampal volume and depression: a meta-analysis of MRI studies. Am J Psychiatry. 161(11):1957–1966. 15514393 10.1176/appi.ajp.161.11.1957

[25] Alexander LM , Escalera J, Ai L, et al 2017. An open resource for transdiagnostic research in pediatric mental health and learning disorders. Sci Data. 4(1):1–26. 10.1038/sdata.2017.181 PMC5735921 29257126

[26] Sandler I , Wolchik S, Braver S, Fogas B. 1991. Stability and quality of life events and psychological symptomatology in children of divorce. Am J Community Psychol. 19(4):501–520. 1755433 10.1007/BF00937989

[27] Frick PJ . 1991. The Alabama parenting questionnaire. Unpublished rating scale, Tuscaloosa (AL): University of Alabama.

[28] Achenbach TM . 2001. Manual for ASEBA school-age forms & profiles. Burlington (VT): University of Vermont, Research Center for Children, Youth & Families.

[29] Hanson JL , Chandra A, Wolfe BL, Pollak SD. 2011. Association between income and the hippocampus. PLoS One 6(5):e18712. 10.1371/journal.pone.0018712 PMC3087752 21573231

[30] Storey JD , Tibshirani R. 2003. Statistical significance for genomewide studies. Proc Natl Acad Sci USA. 100(16):9440–9445. 12883005 10.1073/pnas.1530509100 PMC170937

[31] Ruck MD , Mistry RS, Flanagan CA. 2019. Children's and adolescents’ understanding and experiences of economic inequality: an introduction to the special section. Dev Psychol. 55(3):449–456. 30802097 10.1037/dev0000694

[32] Hair NL , Hanson JL, Wolfe BL, Pollak SD. 2022. Low household income and neurodevelopment from infancy through adolescence. PLoS One 17(1):e0262607. 10.1371/journal.pone.0262607 PMC8791534 35081147

[33] Masten AS , et al 2004. Resources and resilience in the transition to adulthood: continuity and change. Dev Psychopathol. 16(4):1071–1094. 15704828 10.1017/s0954579404040143

[34] Yamaoka Y , Bard DE. 2019. Positive parenting matters in the face of early adversity. Am J Prev Med. 56(4):530–539. 30772146 10.1016/j.amepre.2018.11.018 PMC10954083

[35] Blankenship SL , Chad-Friedman E, Riggins T, Dougherty LR. 2019. Early parenting predicts hippocampal subregion volume via stress reactivity in childhood. Dev Psychobiol. 61(1):125–140. 30288730 10.1002/dev.21788

[36] Morawska A , Dittman CK, Rusby JC. 2019. Promoting self-regulation in young children: the role of parenting interventions. Clin Child Fam Psychol Rev. 22(1):43–51. 30715651 10.1007/s10567-019-00281-5

[37] Hanson JL , Gillmore AD, Yu T, et al 2019. A family focused intervention influences hippocampal-prefrontal connectivity through gains in self-regulation. Child Dev. 90(4):1389–1401. 30295319 10.1111/cdev.13154 PMC6453760

[38] Mistry RS , Elenbaas L. 2021. It's all in the family: parents’ economic worries and youth's perceptions of financial stress and educational outcomes. J Youth Adolesc. 50(4):724–738. 33515373 10.1007/s10964-021-01393-4 PMC7847230

[39] De Los Reyes A , Alfano CA, Lau S, Augenstein TM, Borelli JL. 2016. Can we use convergence between caregiver reports of adolescent mental health to index severity of adolescent mental health concerns? J Child Fam Stud. 25(1):109–123.

[40] Young R , Lennie S, Minnis H. 2011. Children's perceptions of parental emotional neglect and control and psychopathology. J Child Psychol Psychiatry. 52(8):889–897. 21438874 10.1111/j.1469-7610.2011.02390.x PMC3170712

[41] Smith KE , Pollak SD. 2021. Rethinking concepts and categories for understanding the neurodevelopmental effects of childhood adversity. Perspect Psychol Sci. 16(1):67–93. 32668190 10.1177/1745691620920725 PMC7809338

[42] Gee DG . 2020. Caregiving influences on emotional learning and regulation: applying a sensitive period model. Curr Opin Behav Sci. 36:177–184. 33718534 10.1016/j.cobeha.2020.11.003 PMC7945765

[43] Ellis BJ , Sheridan MA, Belsky J, McLaughlin KA. 2022. Why and how does early adversity influence development? Toward an integrated model of dimensions of environmental experience. Dev Psychopathol. 34(2):447–471. 35285791 10.1017/S0954579421001838

[44] Pinquart M . 2017. Associations of parenting dimensions and styles with externalizing problems of children and adolescents: an updated meta-analysis. Dev Psychol. 53(5):873–932. 28459276 10.1037/dev0000295

[45] Vize CE , et al 2022. Moderation effects in personality disorder research. Personal Disord. 14:118–126. 35737564 10.1037/per0000582 PMC9990702

[46] Schmidt AF , Groenwold RH, Knol MJ, et al 2014. Exploring interaction effects in small samples increases rates of false-positive and false-negative findings: results from a systematic review and simulation study. J Clin Epidemiol. 67(7):821–829. 24768005 10.1016/j.jclinepi.2014.02.008

