# Supplementary material for: Positive parenting moderates associations between childhood stress and corticolimbic structure
Source: PNAS Nexus. 2023 Jun 13;2(6):pgad145. doi: 10.1093/pnasnexus/pgad145 (PMC10263262; doi:10.1093/pnasnexus/pgad145)
Supplement: pgad145_Supplementary_Data [file pgad145_supplementary_data.pdf]

Positive parenting moderates associations between childhood stress and corticolimbic structure: supplement

Study GitHub: https://github.com/isabellakahhale/CorticoLimbicParenting

Isabella Kahhale MS, Kelly R. Barry MS, Jamie L Hanson, PhD

04/18/2023

Contents

Supplemental Tables, Figures, & Models 3

Methods 3

Participants . . . 3

Self-Report Measures . . . 3

MRI Site Information, Data Acquisition, Data Processing, and Qualtity Metrics . . . 6

Table S1. Scan Parameters . . . 6

Statistical Analyses . . . 8

Summary Statistics . . . 9

Table S2. Descriptive Statistics Displayed by Site . . . 9

Table S3. Diagnostic Characteristics of HBN Subsample Displayed by Site . . . 10

Table S4. Correlation Table of Key Variables . . . 11

Figure S1: Youth Age Distributions by Sex . . . 12

Main Manuscript Models: Regression Diagnostics & Simpler Models without Interaction Terms . . 13

Regression Diagnostics for Main Manuscript Models . . . 13

Main Manuscript Models without Interaction Term . . . 18

Examining Associations Between Youth Behavioral Problems, Stress, Parenting, and an Interaction of Stress X Parenting . . . 23

Caregiver-Report of Youth Behavioral Problems . . . 23

Socioeconomic Status (SES) Variables . . . 25

Correlations between Stress and Multiple Operationalizations of SES . . . 25

Correlations between Parenting and Multiple Operationalizations of SES . . . 25

Sensitivity Analysis with Socioeconomic Status (SES) as an Additional Variable in Main Models 26

Cognitive Ability as a Covariate in Main Analyses . . . 35

Correlations between stress and IQ . . . 35

IQ as a covariate in main analyses . . . 36
Sensitivity Analysis with Psychopathology Diagnosis as an Additional Variable in Main Models . . 39
Probing Questions of Interest Using Generalized Additive Mixed Models (GAMMs) to Account for Potential Non-Linear Age Effects . . . 42
Probing Questions of Interest Using a Quadtratic Stress Term to Account for Potential Non-Linear Stress Effects . . . 49
Investigating Total Brain Region Volumes with Total Grey Matter Volume as an Alternative Covariate to Estimated Total Intracranial Volume . . . 56
Investigating Left and Right Hemispheres of Brain Regions Separately . . . 61
Examining Associations Between Hippocampal Volumes and Youth Behavioral Problems . . . 70
Comparing Key Variables between HBN-Imaging Sample to HBN-Total Sample . . . 77

Supplemental References 80

Supplemental Tables, Figures, & Models

Methods

All code used for data cleaning and analysis, as well as a list of the HBN subjects used for this project, can be found on our study GitHub.

Participants

Data from 482 participants (39% female, 61% male) between the ages of 10-17 with T1-weighted structural images were downloaded from an ongoing research initiative, the Healthy Brain Network (HBN), launched by The Child Mind Institute in 2015. Participants with cognitive or behavioral challenges (e.g., being nonverbal, IQ<66), or with serious medical concerns were excluded from the HBN project. For sample characteristics, see Table 1. In terms of racial and ethnic identities, 241 participants identified as White (50%), 64 identified as Black/African American (13.3%), 45 identified as Hispanic (9.3%), 10 identified as Asian (2.1%), 7 identified as Indian (1.5%), 82 (17%) identified as Bi- or Mult-iracial, 9 identified as Other (1.9%; e.g., Middle Eastern), and 24 chose "unknown" or chose not to specify their race (5%). For additional information about the HBN sample, please see the HBN data-descriptor. Internal consistency estimates for each questionnaire measure appear in the Supplement and range from "good" to "excellent" (Chronbach's alpha = 0.706-0.900).

Self-Report Measures

Negative Life Events Scale

We operationalized childhood stress by using the Negative Life Events Scale (NLES). Example items from the NLES include: "You have suffered from a serious physical illness, injury, or extreme pain", "Your mom or dad was arrested or sent to jail", and "His/her relatives said bad things about his/her mother or father." Caregivers completed the same scale reporting on the negative life events their child may or may not have experienced. Previous work has found that caregiver and youth reports of negative life events are correlated (r = 0.49-0.72), although children tend to report a greater number of negative life events (Johnston, 2003). In this supplement, we consider analyses with both youth-reported NLES and caregiver-reported NLES scores in analyses.

Internal reliability estimates were calculated both for the full NLE (Youth Report) and the "Average Upsetness" Score (Youth Report) we used as our main outcome variable, using the total sample of 485 observations. Chronbach's alpha values were within the "good" to "excellent" range, with Chronbach's *alpha* for full NLE (Youth) = 0.9 and Chronbach's *alpha* for "Average Upsetness" Score (Youth) = 0.766.

Internal reliability estimates were calculated both for the full NLE (Parent Report) and the "Average Upsetness" Score (Parent Report) we used as our main outcome variable, using the total sample of 485 observations. Chronbach's alpha values were within the "acceptable" to "good" range, with Chronbach's *alpha* for full NLE (Parent) = 0.87 and Chronbach's *alpha* for "Average Upsetness" Score (Parent) = 0.706.

Alabama Parenting Questionnaire: Positive Parenting Subscale

The full APQ is a 42-item measure designed to assess dimensions of parenting (e.g., inconsistent discipline, corporal punishment, poor monitoring/supervision, positive parenting) strongly correlated with child behavior (Dadds et al., 2003; Shelton et al., 1996). The positive parenting subscale (APQ-PP) is the sum of 6 items (e.g., " You let your child know when he/she are doing a good job with something"). The APQ subscales demonstrate favorable reliability and convergent validity in both youth (Essau et al., 2006; Liang et al., 2021). Internal reliability estimates were calculated both for the full APQ (Youth Report) and the Positive Parenting Subscale (Youth Report) using the total sample of 485 observations. Chronbach's alpha

values were within the "good" range, with Chronbach's *alpha* for full APQ (Youth) = 0.875 and Chronbach's *alpha* for Positive Parenting Subscale (Youth) = 0.815. Internal reliability estimates were also calculated both for the full APQ (Parent Report) and the Positive Parenting Subscale (Parent Report) using the total sample of 485 observations. Chronbach's alpha values were within the "good" range, with Chronbach's *alpha* for full APQ (Parent) = 0.855 and Chronbach's *alpha* for Positive Parenting Subscale (Parent) = 0.843.

Youth Self-Report

We operationalized youth problem behavior in two ways, the first being through the Youth Self-Report Total Problems Raw Score. Studies of the YSR's psychometric properties report discriminant and convergent validity among clinical, non-clinical, and diverse samples and acceptable reliability metrics (e.g., mean test-retest reliability = 0.79, mean internal consistency = 0.83) (Achenbach et al., 1995). We used the Total Problems Raw Score over the T-score due to literature advising Raw YSR scores to be used in statistical analyses. There is evidence that T scores truncate variability, and that conclusions based on these truncated scores are less reliable (Thurber & Sheehan 2012). The creators of the YSR also advocate for using raw scores in data analyses (Achenbach & Rescorla, 2001).

Internal reliability estimates were calculated for the Youth Self Report Total Raw score using the total sample of 485 observations. Chronbach's alpha values were well within the "excellent" range with Chronbach's *alpha* for YSR Total score = 0.993.

Strengths & Difficulties Questionnaire

We also considered an alternative measure of youth behavioral problems, the Strengths and Difficulties Questionnaire (SDQ). Specifically, we used SDQ Ttal Difficulties Score as an alternative, caregiver-reported operationalization. The SDQ is a well-validated and widely used instrument with acceptable psychometric properties (i.e., average test-retest reliability = 0.72; average internal consistency = 0.71, average inter-rater reliability = 0.39) (Goodman, 2001). In the HBN Battery, the 30-item questionnaire was completed by caregivers. The SDQ the following subscales and total score: 1) Emotional Symptoms, 2) Conduct Problems, 3) Hyperactivity/Inattention, 4) Peer Problems, 5) Prosocial Behavior, and 6) a Total Difficulties Score. For additional information on the SDQ, including psychometric properties and language translations, please see: https://www.nctsn.org/measures/strengths-and-difficulties-questionnaire-parent-report.

Internal reliability estimates were calculated for the Strength and Difficulties Questionnaire (SDQ) Total Difficulties score using the total sample of 485 observations. Chronbach's alpha values were well within the "good" range with Chronbach's *alpha* for SDQ Total Difficulties score = 0.884.

Financial Support Questionnaire (FSQ)

The Financial Support Questionnaire (FSQ) is a measured developed by the Child Mind Institute/Healthy Brain network and measures a variety of socioeconomic-related parameters, including employment status of primary caregiver (i.e., the caregiver at the study visit; FSQ_02; 0 = unemployed, 1 = employed) and employment status of the child's second caregiver (FSQ_03; 0 = unemployed, 1 = employed, 2 = no second primary caregiver). We recoded the second-caregiver employment variable (FSQ_03) so that a response of "no second primary caregiver" was given the value "0". We then summed values from primary caregiver employment and secondary caregiver employment for an overall variable representing parental employment (ie.., 0 = no caregiver employment, 1 = one caregiver is employed, 2 = two caregivers are employed). From the FSQ, we also considered Income (i.e., "what is your annual household income?") and recoded the categorical Income variable by multiplying each value by the midpoint of each income range bracket and then taking the natural log.

Barratt Simplified Measure of Social Status (BSMSS)

The Barratt Simplified Measure of Social Status (BSMSS) is a measure of "Social Status" based on the work of Hollingshead (1957, 1975). This measure considers caregiver education level/attainment and occupational

“prestige.” In analyses considering socioeconomic status as a covariate, we used the Barratt Total score. We also constructed a composite variable that a) selects the higher of the two occupations and education levels between two caregivers (and considers the occupation and education level of the single caregiver, if applicable), b) mean-centered each variable (i.e., took the “z-score”), and c) derived the mean of the two.

Wechsler Intelligence Scale for Children-V (WISC-V)

The Wechsler Intelligence Scale for Children-V (WISC-V) is a widely used test that provides a global index of cognitive performance. The main variable we considered as a measure of intelligence the Full Scale Intelligence Quotient (FSIQ), or a numerical representation of a child’s intellectual ability.

MRI Site Information, Data Acquisition, Data Processing, and Qualtity Metrics

The HBN project conducted MRI scans at 4 different sites: Citigroup Biomedical Imaging Center, the City University of New York, Rutgers University, and Staten Island. Eighty-two participants were scanned at the HBN Diagnostic Research Center in Staten Island (SI), 200 scanned at Rutgers University Brain Imaging Center (RU), 186 scanned at the CitiGroup Cornell Brain Imaging Center (CBIC), and 14 scanned at the City University of New York (CUNY).

Table S1 depicts the scanning parameters for each scanner. These scans were collected on different MRI scanners, specifically a 1.5 T Siemens Avanto scanner at SI, a Siemens 3T Tim Trio scanner at RU, Siemens 3T Prisma scanner at RU and CBIC, and a Siemens 3T Prisma scanner at CUNY. All structural MRI scans were 3D T1-weighted sequences and specific scan sequence parameters are included in the table below. As discussed in the manuscript, we used linear mixed effects models to account for (MRI-related) site variations given differences in MRI acquisition. Of note, all neuroimaging data used in this study are openly available for download with proper data usage agreement via the International Neuroimaging Data-sharing Initiative (fcon_1000.projects.nitrc.org/indi/cmi_healthy_brain_network/).

Table S1. Scan Parameters

Study Site	Scanner Details	Slices	Resolution (mm)	TR (ms)	TE (ms)	Flip Angle (deg)	Type
RU	3T Siemens Magnetom Trio Tim	224	.8 x .8 x .8	2500	3.15	8	Non-sel. IR
SI	1.5T Siemens Avanto	176	1 x 1 x 1	2730	1.64	7	Non-sel. IR
CBIC	3T Siemens Prisma	224	.8 x .8 x .8	2500	3.15	8	Non-sel. IR
CUNY	3T Siemens Prisma	224	.8 x .8 x .8	2500	3.15	8	Non-sel. IR

CBIC = Citigroup Biomedical Imaging Center, CUNY = The City University of New York, RU = Rutgers University, SI = Staten Island

Table S1. Scanning parameters for each scanner used in the HBN Study. Data obtained from all four scanners are used in this project.

MRI Data Processing

Freesurfer is a widely documented and freely available morphometric processing tool suite (http://surfer.nmr.mgh.harvard.edu/) The technical details of these procedures are described in prior publications (Dale et al., 1999; Fischl et al., n.d., 2002, 2004; Fischl, Sereno, & Dale, 1999; Fischl, Sereno, Tootell, et al., 1999). Briefly, this processing includes motion correction and intensity normalization of T1-weighted images, removal of non-brain tissue using a hybrid watershed/surface deformation procedure (Fischl et al., 2004), automated Talairach transformation, segmentation of the subcortical white matter and deep gray matter volumetric structures (including hippocampus, amygdala, caudate, putamen, ventricles), tessellation of the gray matter white matter boundary, and derivation of cortical surface area and cortical thickness. Of note, the "recon-all" pipeline with the default set of parameters (no flag options) was used and no manual editing was conducted. In keeping with our past work (Gilmore et al., 2021), Freesurfer outputs were checked via research staff for major errors, and via automated methods (see next section). After successful processing and quality assurance, we extracted volumes for our subcortical structures of interest– the hippocampus and amygdala. We calculated the total volume (for the hippocampus or amygdala) by summing volumes from the left and right hemispheres of each structure. Freesurfer was implemented using Brainlife.io, (brainlife.app.0, https://doi.org/10.25663/bl.app.0), which is a free, publicly funded, cloud-computing platform for reproducible neuroimaging pipelines and data sharing (Avesani et al., 2019), for additional information, visit http://brainlife.io/).

Image Quality Metrics

We assessed image quality to exclude particularly high-motion scans and limit the impact of image quality on subcortical volume quantification. Past work from our group (e.g., Gilmore et al., 2021) has found that T1-weighted image quality is related to volumetric measures from commonly used morphometric tools suites (e.g., Freesurfer). We therefore assessed image quality to: 1) Exclude particularly high-motion scans; and 2) limit the impact of image quality on subcortical volume quantification. To assess MRI quality, we generated a quantitative metric ("CAT12 score") using the Computational Anatomy Toolbox 12 (CAT12) (Gaser et al., 2016). This metric considers four summary measures of image quality: noise-to-contrast ratio, coefficient of joint variation, inhomogeneity-to-contrast ratio, and root-mean-squared voxel resolution. CAT12 normalizes and combines these measures using a kappa statistic-based framework. The score is a value from 0 to 1, with higher values indicating better image quality. Additional information is available at: http://www.neuro.uni-jena.de/cat/index.html#QA.

Statistical Analyses

Here, we elaborate on the analytic plan from our manuscript in further detail. We first fit ordinary least squares regression models to examine the interactions of stress and parenting in predicting youth behavioral problems. This involved entering the total scores from the YSR as the dependent variable, and sex, age, site, youth-reported negative life events, youth-reported positive parenting, and an interaction between youth-reported negative life events and youth-reported positive parenting as independent variables. All continuous variables were mean-centered.

Next, to model relations between variables of interest and deal with potential variations in research sites, we fit linear mixed-effects models (LMEM) using the R package 'lmer' (Bates et al., 2015). For each brain region of interest (i.e., total hippocampal volume, total amygdala volume), we first fit a LMEM with a random effect of site, main effects of sex, age, estimated total intracranial volume, youth-reported negative life events, youth-reported positive parenting, and an interaction between negative life events and youth-reported positive parenting. This was motivated by our interest in youth perceptions of parenting behaviors. Notably, we also fit a second set of LMEMs for each brain region of interest again including a random effect of site, main effects of sex, age, eTIV, and youth-reported negative life events, this time including caregiver-reported positive parenting and an interaction between youth-reported negative life events and caregiver-reported positive parenting. This was to examine whether caregiver perceptions of positive parenting had different associations with brain volumes (compared to youth perceptions). All continuous variables were mean-centered for these analyses. For all significant interaction terms, we then conducted follow-up analyses of the simple slopes of subgroups (the mean of all individuals, +1 SD of the mean, and -1 SD of the mean) using the "interactions" R library (Long, 2019). R code and output for all analyses are included in this document.

Across models, we also tested for differences between any significant (and non-significant) interaction terms. This was done when comparing the interaction results for youth-reported negative life events and youth-reported positive parenting (in predicting structural volumes) to interaction results for youth-reported negative life events and caregiver-reported positive parenting. For such comparisons, we used Williams's Test, a statistical comparison used when two correlation coefficients are calculated from a single sample, may be collinear, and not statistically independent (Dunn & Clark, 1971; Williams, 1971). This was done using the "psych" package in R (Revelle, 2022).

Additional sensitivity analyses (presented in the supplemental materials) examine both caregiver and youth reports of negative life events, positive parenting, and youth behavior within our main models; main effect models without interaction terms; four different operationalization of socioeconomic status; the contribution of psychopathology and cognitive ability in smaller overall samples (n = 226 and n = 320, respectively); questions of interest using General Additive Mixed Models (GAMMs) to account for non-linear age effects; models of stress with a non-linear quadratic term in LMEMs and GAMMs; total gray matter volume as an alternative brain scaling variable; left and right hemispheres of brain regions separately; associations between hippocampal volumes and youth behavioral problems; and comparisons of key variables between the HBN imaging sample anad the total HBN sample.

Summary Statistics

Table S2. Descriptive Statistics Displayed by Site

Table S2. Descriptive Statistics for Key Variables

data reported by site

Characteristic	CBIC[1]	CUNY[1]	RU[1]	SI[1]	Overall[1]
Total	N = 186	N = 14	N = 200	N = 82	N = 482
Age	13.59 (2.04)	13.09 (2.04)	13.20 (2.46)	13.17 (3.07)	13.34 (2.42)
Sex					
Female	75 (40%)	3 (21%)	73 (36%)	39 (48%)	190 (39%)
Male	111 (60%)	11 (79%)	127 (64%)	43 (52%)	292 (61%)
Income					
Less than $10,000	3 (1.6%)	0 (0%)	7 (3.5%)	0 (0%)	10 (2.4%)
$10,000 to $19,999	3 (1.6%)	0 (0%)	6 (3.0%)	2 (9.5%)	11 (2.6%)
$20,000 to $29,999	7 (3.8%)	0 (0%)	5 (2.5%)	0 (0%)	12 (2.9%)
$30,000 to $39,999	12 (6.6%)	0 (0%)	4 (2.0%)	0 (0%)	16 (3.8%)
$40,000 to $49,999	4 (2.2%)	1 (7.1%)	12 (6.1%)	1 (4.8%)	18 (4.3%)
$50,000 to $59,999	4 (2.2%)	0 (0%)	3 (1.5%)	4 (19%)	11 (2.6%)
$60,000 to $69,999	6 (3.3%)	0 (0%)	10 (5.1%)	0 (0%)	16 (3.8%)
$70,000 to $79,999	4 (2.2%)	0 (0%)	7 (3.5%)	1 (4.8%)	12 (2.9%)
$80,000 to $89,999	12 (6.6%)	2 (14%)	12 (6.1%)	0 (0%)	26 (6.2%)
$90,000 to $99,999	5 (2.7%)	1 (7.1%)	14 (7.1%)	0 (0%)	20 (4.8%)
$100,000 to $149,999	36 (20%)	3 (21%)	38 (19%)	4 (19%)	81 (19%)
$150,000 or more	62 (34%)	7 (50%)	58 (29%)	1 (4.8%)	128 (31%)
Choose not to disclose	25 (14%)	0 (0%)	22 (11%)	8 (38%)	55 (13%)
Positive Parenting: Youth	21 (4)	21 (6)	21 (5)	23 (5)	21 (5)
Positive Parenting: Parent	25 (3)	26 (3)	25 (3)	25 (3)	25 (3)
Negative Life Events: Youth	2.85 (1.15)	2.91 (1.11)	2.84 (1.19)	2.82 (1.21)	2.84 (1.17)
Negative Life Events: Caregiver	2.66 (0.91)	1.77 (0.94)	2.51 (1.03)	2.31 (1.15)	2.52 (1.01)
Behavioral Problems: Youth	48 (25)	44 (19)	49 (28)	52 (23)	48 (26)
Behavioral Problems: Caregiver	11 (6)	13 (7)	12 (6)	12 (7)	11 (6)
Total Gray Matter Volume[2]	758 (76)[2]	791 (49)[2]	737 (74)[2]	726 (78)[2]	745 (76)[2]

[1] N = N; Mean (SD); n (%)

[2] Total gray matter volume values are divided by 1,000

CBIC = Citigroup Biomedical Imaging Center, CUNY = The City University of New York, RU = Rutgers University, SI = Staten Island. Units of measurement are the following: Age (years), Positive Parenting: Youth/Caregiver (raw scores from the Alabama Parenting Questionnaire: Positive Parenting Subscale, Negative Life Events: Youth/Caregiver (Negative Life Events Scale, average upsetness score), Behavioral Problems: Youth (Youth Self Report Total Behavioral Problems Raw Score), Behavioral Problems: Caregiver (Strength and Difficulties Questionnaire Total Raw Score), and Total Gray Matter Volume (voxels divided by 1,000).

Table S2. Summary statistics for key study variables displayed by imaging site.

Table S3. Diagnostic Characteristics of HBN Subsample Displayed by Site

Table S3. Psychopathology data for HBN participants

data reported by site

Characteristic	CBIC[1]	RU[1]	SI[1]	Overall[1]
Total	N = 52	N = 92	N = 82	N = 226
Age	13.63 (2.02)	12.63 (2.50)	13.17 (3.07)	13.06 (2.65)
Sex				
Female	22 (42%)	36 (39%)	39 (48%)	97 (43%)
Male	30 (58%)	56 (61%)	43 (52%)	129 (57%)
Any DSM-5 Diagnosis				
No Dx	4 (7.7%)	13 (14%)	22 (27%)	39 (17%)
Yes Dx	48 (92%)	79 (86%)	60 (73%)	187 (83%)
First Diagnosis Category				
Anxiety Disorders	7 (13%)	10 (11%)	12 (15%)	29 (13%)
Depressive Disorders	5 (9.6%)	11 (12%)	12 (15%)	28 (12%)
Disruptive Disorders	0 (0%)	3 (3.3%)	2 (2.4%)	5 (2.2%)
Neurodevelopmental Disorders	34 (65%)	52 (58%)	30 (37%)	116 (52%)
Obsessive Compulsive and Related Disorders	1 (1.9%)	1 (1.1%)	1 (1.2%)	3 (1.3%)
Schizophrenia Spectrum and other Psychotic Disorders	0 (0%)	0 (0%)	1 (1.2%)	1 (0.4%)
Trauma and Stressor Related Disorders	0 (0%)	0 (0%)	0 (0%)	0 (0%)
Other Conditions That May Be a Focus of Clinical Attention	1 (1.9%)	0 (0%)	2 (2.4%)	3 (1.3%)
No Diagnosis Given	4 (7.7%)	13 (14%)	22 (27%)	39 (17%)
Second Diagnosis Category				
Anxiety Disorders	13 (25%)	11 (12%)	7 (8.6%)	31 (14%)
Bipolar and Related Disorders	0 (0%)	0 (0%)	1 (1.2%)	1 (0.4%)
Depressive Disorders	1 (1.9%)	4 (4.3%)	4 (4.9%)	9 (4.0%)
Disruptive Disorders	1 (1.9%)	5 (5.4%)	1 (1.2%)	7 (3.1%)
Elimination Disorders	2 (3.8%)	1 (1.1%)	0 (0%)	3 (1.3%)
Neurodevelopmental Disorders	19 (37%)	27 (29%)	15 (19%)	61 (27%)
Obsessive Compulsive and Related Disorders	0 (0%)	1 (1.1%)	1 (1.2%)	2 (0.9%)
Trauma and Stressor Related Disorders	0 (0%)	0 (0%)	0 (0%)	0 (0%)
No Secondary Diagnosis	16 (31%)	43 (47%)	52 (64%)	111 (49%)

[1] N = N; Mean (SD); n (%)

CBIC = Citigroup Biomedical Imaging Center, RU = Rutgers University, SI = Staten Island.

Table S3. Psychopathology characteristics of a sub-sample of HBN participants displayed by imaging site.

Table S4. Correlation Table of Key Variables

This correlation table can also be viewed on GitHub.

Correlation Table

Means, standard deviations, and correlations with confidence intervals

Variable	*M*	*SD*	1	2	3	4	5	6	7	8	9	10	11
1. Age	13.34	2.42											
2. Income	4.98	0.37	.13*<br>[.03, .23]										
3. Childhood Stress (NLE Youth Report)	2.84	1.17	.11*<br>[.02, .20]	-0.03<br>[-.14, .07]									
4. Childhood Stress (NLE Caregiver Report)	2.52	1.01	.10*<br>[.00, .19]	0<br>[-.10, .11]	.15**<br>[.06, .24]								
5. Positive Parenting (Youth Report)	21.4	4.93	-.21**<br>[-.29, -.12]	-0.05<br>[-.16, .05]	0.05<br>[-.04, .14]	0.01<br>[-.08, .10]							
6. Positive Parenting (Caregiver Report)	24.98	3.16	-.21**<br>[-.29, -.12]	-.12*<br>[-.22, -.02]	-0.03<br>[-.12, .06]	0<br>[-.09, .10]	.22**<br>[.13, .30]						
7. Behavioral Problems (YSR/Youth Report)	48.48	25.91	.16**<br>[.06, .25]	-.13*<br>[-.24, -.02]	.13**<br>[.03, .22]	.11*<br>[.01, .21]	-.24**<br>[-.33, -.14]	-0.02<br>[-.12, .08]					
8. Behavioral Problems (SDQ/Caregiver Report)	11.18	6.36	-0.09<br>[-.19, .00]	-.22**<br>[-.32, -.11]	0.01<br>[-.08, .11]	0.1<br>[-.00, .20]	0.04<br>[-.06, .14]	0.08<br>[-.01, .18]	.24**<br>[.14, .33]				
9. Total Hippocampal Volume	8368.45	949.79	.14**<br>[.05, .22]	.18**<br>[.08, .28]	-0.07<br>[-.16, .01]	0.06<br>[-.03, .15]	-.13**<br>[-.21, -.04]	0.03<br>[-.06, .12]	0.02<br>[-.08, .12]	0<br>[-.10, .09]			
10. Total Amygdala Volume	3480.67	441.98	.15**<br>[.06, .23]	0.1<br>[-.00, .20]	-0.06<br>[-.14, .03]	0.06<br>[-.03, .15]	-.16**<br>[-.24, -.07]	-0.02<br>[-.11, .07]	0.05<br>[-.05, .14]	-0.02<br>[-.12, .07]	.74**<br>[.70, .78]		
11. Total Intracranial Volume	1527636.9	178891.58	.10*<br>[.01, .19]	.11*<br>[.01, .21]	-0.04<br>[-.13, .05]	-0.03<br>[-.12, .07]	-.11*<br>[-.19, -.02]	0.02<br>[-.07, .11]	0.04<br>[-.06, .14]	0.06<br>[-.04, .16]	.65**<br>[.59, .70]	.60**<br>[.54, .65]	
12. Scan Quality	0.87	0.02	.10*<br>[.01, .19]	0<br>[-.10, .11]	0.05<br>[-.04, .14]	0<br>[-.09, .10]	-.15**<br>[-.24, -.06]	-.10*<br>[-.19, -.01]	0.06<br>[-.04, .16]	0.06<br>[-.04, .15]	.21**<br>[.13, .30]	.23**<br>[.14, .31]	0<br>[-.09, .09]

Note. *M* and *SD* are used to represent mean and standard deviation, respectively. Values in square brackets indicate the 95% confidence interval for each correlation. The confidence interval is a plausible range of population correlations that could have caused the sample correlation (Cumming, 2014). * indicates p < .05. ** indicates p < .01.

Table S4. M and SD are used to represent mean and standard deviation, respectively. Values in square brackets indicate the 95% confidence interval for each correlation. The confidence interval is a plausible range of population correlations that could have caused the sample correlation. * indicates p < .05. ** indicates p < .01.

Figure S1: Youth Age Distributions by Sex

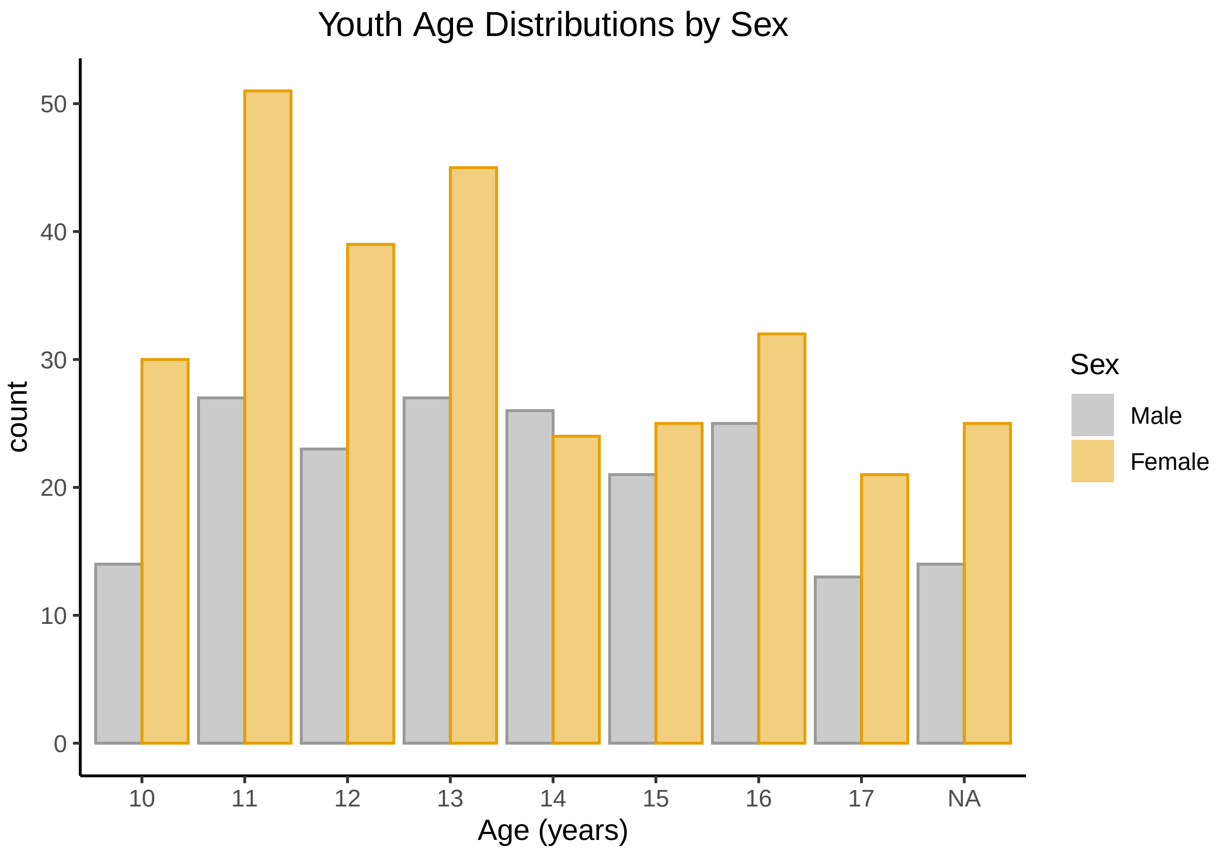

Figure S1. Youth participant age distributions by biological sex.

We computed a two-sample Z-test to analyze a potential difference among the two distributions (i.e., the distributions of age after splitting the sample by biological sex). The Z-test was non-significant (Z-statistic = -1.482, p = 0.138) indicating that the age distributions do not significantly differ by sex.

Main Manuscript Models: Regression Diagnostics & Simpler Models without Interaction Terms

Regression Diagnostics for Main Manuscript Models

We examined a variety of regression diagnostics including outlier values, QQ plots for studentized residuals, and leverage plots for our main models below (i.e., childhood stress and youth-report parenting predicting youth behavioral problems; childhood stress and youth-report parenting predicting total hippocampal volumes).

Regression Diagnostics: Childhood stress and youth-report parenting on youth-report behavioral problems

Examination of outlier values, QQ plots for studentized residuals, and leverage plots revealed two points that may be influential in the model considering the interaction between (youth report) stress and (youth report) parenting on (youth report) behavioral problems.

Model Output. This table depicts linear regression model output, including standardized regression coefficients (Est.), standard errors (S.E.), t-values (t val.), and p-values (p-val). Model output also depicts overall model fit statistics and number of observations.

```
##     rstudent unadjusted p-value Bonferroni p
## 317 4.293203          2.2211e-05    0.0089734
## 22  4.265711          2.4993e-05    0.0100970
```

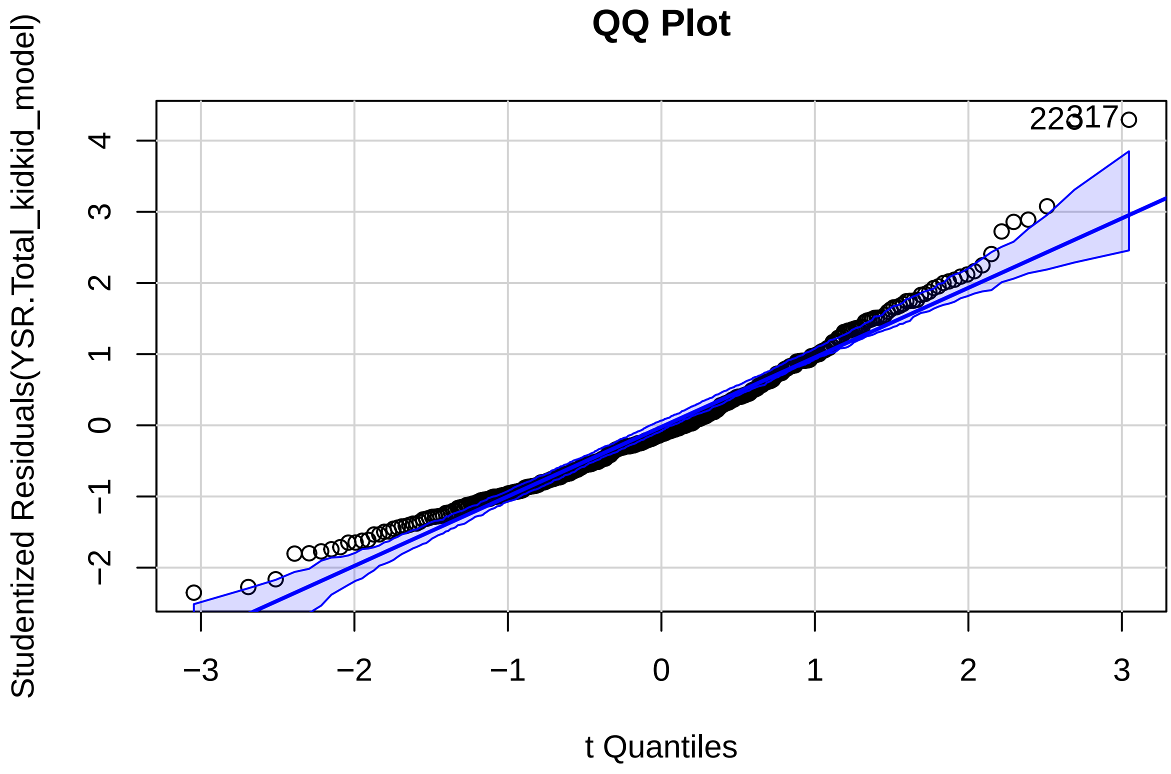


```
## [1]  22 317
```

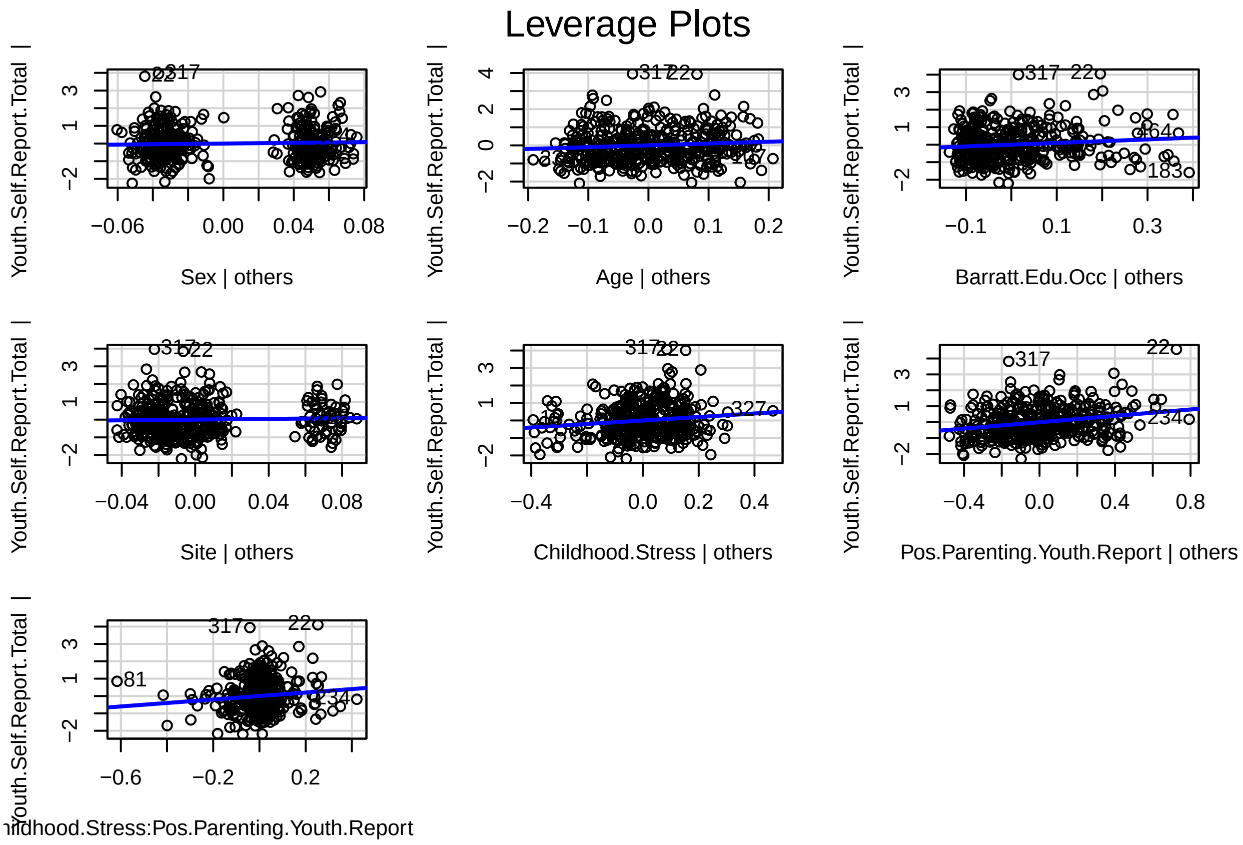

Observations	402 (78 missing obs. deleted)
Dependent variable	Youth.Self.Report.Total
Type	OLS linear regression

F(9,392)	4.7568
R^2	0.0985
Adj. R^2	0.0778

	Est.	S.E.	t val.	p
(Intercept)	-0.0078	0.0905	-0.0865	0.9311
SexMale	-0.1291	0.0951	-1.3581	0.1752
Age	0.1008	0.0575	1.7517	0.0806
Barratt.Edu.Occ	-0.0914	0.0505	-1.8117	0.0708
SiteCUNY	0.0158	0.2740	0.0577	0.9540
SiteRU	0.0302	0.1010	0.2993	0.7649
SiteSI	0.1297	0.1455	0.8909	0.3735
Childhood.Stress	0.1174	0.0483	2.4316	0.0155
Pos.Parenting.Youth.Report	-0.2219	0.0492	-4.5117	0.0000
Childhood.Stress:Pos.Parenting.Youth.Report	-0.0747	0.0451	-1.6539	0.0990

Standard errors: OLS

Model Output. This table depicts linear regression model output, including standardized regression coefficients (Est.), standard errors (S.E.), t-values (t val.), and p-values (p-val). Model output also depicts overall

model fit statistics and number of observations.

Regression Diagnostics: Childhood stress, youth-report parenting, and hippocampal volumes

Model Output. This table depicts mixed effects linear regression model output, including both fixed effects and random effects. Fixed effects include standardized regression coefficients (Est.), standard errors (S.E.), t-values (t val.), and p-values (p-val). Random effects include the random effect of Imaging Site and the number of grouping variables (4 imaging sites). Model output also depicts overall model fit statistics and number of observations.

```
## No Studentized residuals with Bonferroni p < 0.05
## Largest |rstudent|:
##      rstudent unadjusted p-value Bonferroni p
## 411 -3.650907         0.00029112      0.13828
```

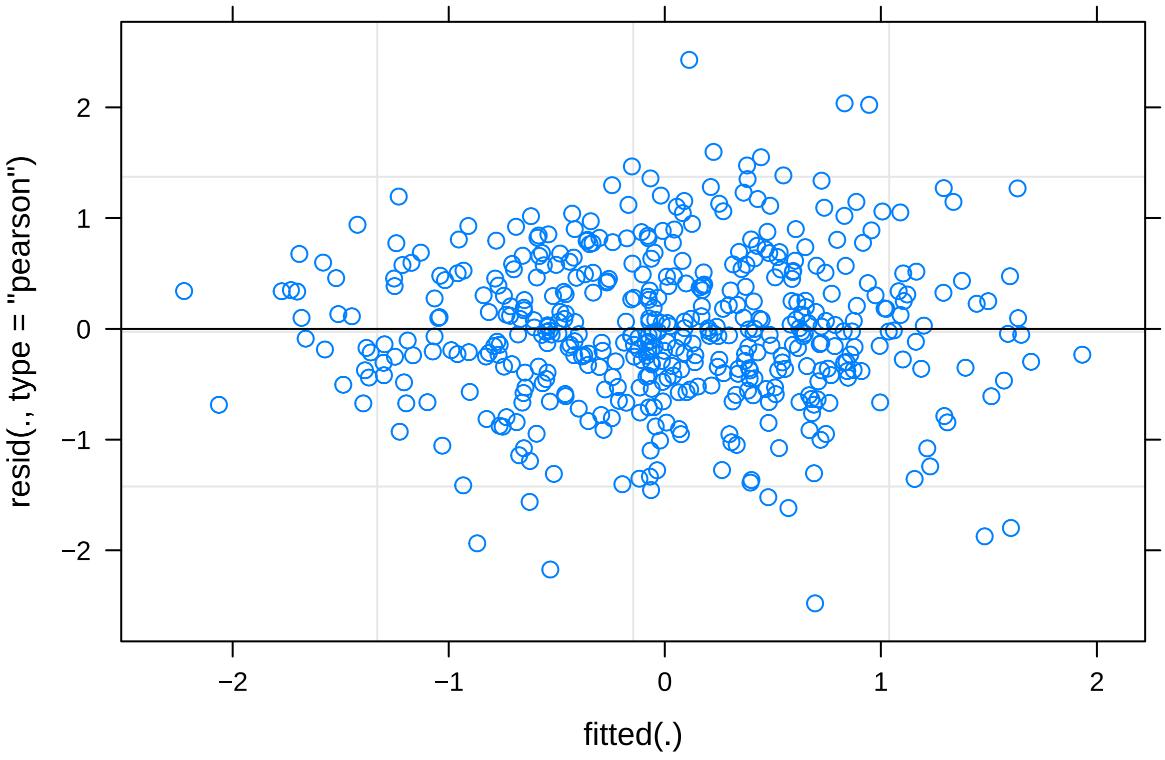

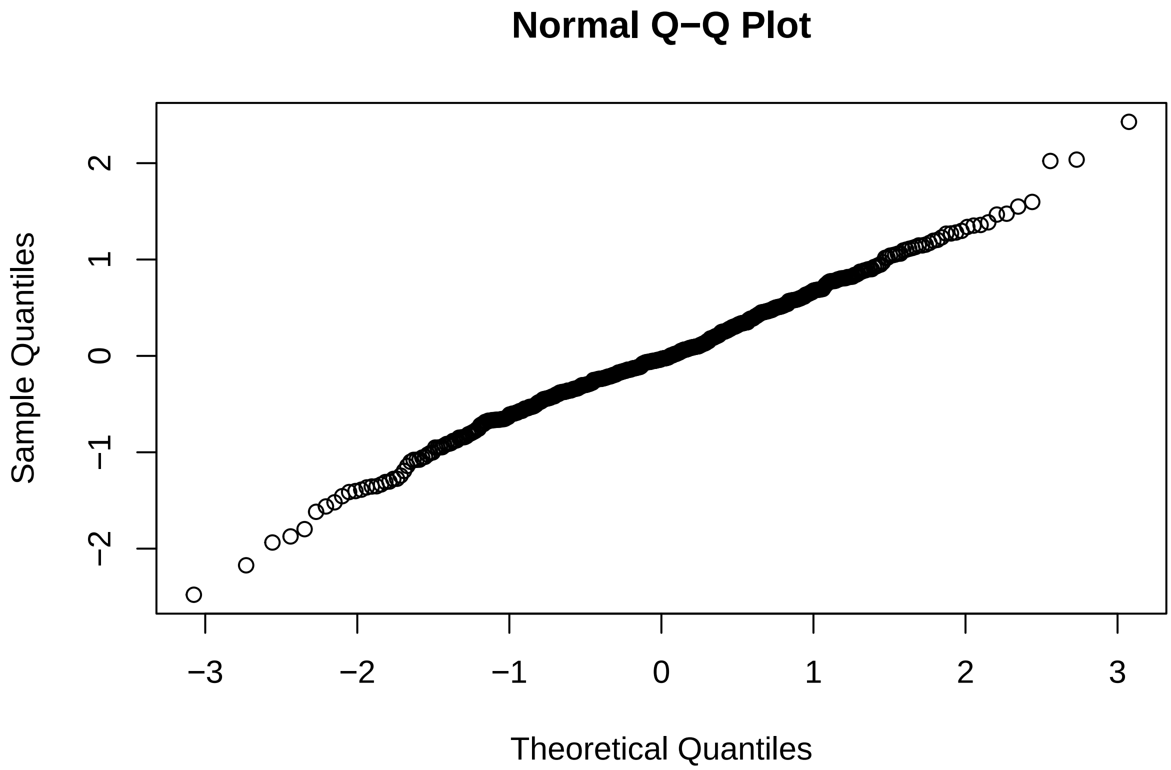

Normal Q−Q Plot
Sample Quantiles
−2
−1
0
1
2
−3
−2
−1
0
1
2
3
Theoretical Quantiles

Observations	474
Dependent variable	TotalHippocampalVolume
Type	Mixed effects linear regression

AIC	1037.2620
BIC	1083.0353
Pseudo-R^2 (fixed effects)	0.4277
Pseudo-R^2 (total)	0.5507

Fixed Effects					
	Est.	S.E.	t val.	d.f.	p
(Intercept)	-0.1433	0.1888	-0.7593	3.4292	0.4965
Scan.Quality	0.0849	0.0384	2.2132	457.8237	0.0274
SexMale	0.1274	0.0756	1.6845	462.5046	0.0928
Age	0.0682	0.0324	2.1065	462.5710	0.0357
Barratt.Edu.Occ	0.0603	0.0338	1.7838	463.0529	0.0751
Total.Intracranial.Vol	0.5933	0.0370	16.0157	463.0427	0.0000
Childhood.Stress	-0.0685	0.0322	-2.1300	462.1698	0.0337
Pos.Parenting.Youth.Report	0.0120	0.0326	0.3678	462.2715	0.7132
Childhood.Stress:Pos.Parenting.Youth.Report	0.0678	0.0301	2.2522	462.9665	0.0248

p values calculated using Satterthwaite d.f.

Random Effects		
Group	Parameter	Std. Dev.
Site	(Intercept)	0.3529
Residual		0.6743

Grouping Variables		
Group	# groups	ICC
Site	4	0.2150

Model Output. This table depicts mixed effects linear regression model output, including both fixed effects and random effects. Fixed effects include standardized regression coefficients (Est.), standard errors (S.E.), t-values (t val.), and p-values (p-val). Random effects include the random effect of Imaging Site and the number of grouping variables (4 imaging sites). Model output also depicts overall model fit statistics and number of observations.

Main Manuscript Models without Interaction Term

Childhood Stress and Youth-Report Parenting on Youth-Report Behavioral Problems

Observations	404 (78 missing obs. deleted)
Dependent variable	Youth.Self.Report.Total
Type	OLS linear regression

F(8,395)	5.7108
R^2	0.1037
Adj. R^2	0.0855

	Est.	S.E.	t val.	p
(Intercept)	-0.0019	0.0947	-0.0205	0.9837
SexMale	-0.1008	0.0994	-1.0141	0.3111
Age	0.1075	0.0603	1.7831	0.0753
Barratt.Edu.Occ	-0.1211	0.0526	-2.3024	0.0218
SiteCUNY	-0.0451	0.2862	-0.1577	0.8748
SiteRU	0.0133	0.1055	0.1259	0.8999
SiteSI	0.1046	0.1526	0.6853	0.4935
Childhood.Stress	0.1291	0.0504	2.5619	0.0108
Pos.Parenting.Youth.Report	-0.2394	0.0507	-4.7197	0.0000

Standard errors: OLS

Model Output. This table depicts linear regression model output, including standardized regression coefficients (Est.), standard errors (S.E.), t-values (t val.), and p-values (p-val). Model output also depicts overall model fit statistics and number of observations.

Childhood Stress and Youth-Report Parenting on Total Hippocampal Volume

Observations	475
Dependent variable	TotalHippocampalVolume
Type	Mixed effects linear regression

AIC	1049.2607
BIC	1090.8939
Pseudo-R^2 (fixed effects)	0.4135
Pseudo-R^2 (total)	0.5386

Fixed Effects					
	Est.	S.E.	t val.	d.f.	p
(Intercept)	-0.1413	0.1912	-0.7390	3.4123	0.5075
Scan.Quality	0.0823	0.0389	2.1130	459.9530	0.0351
SexMale	0.1307	0.0768	1.7025	464.5596	0.0893
Age	0.0677	0.0329	2.0549	464.5385	0.0404
Barratt.Edu.Occ	0.0628	0.0342	1.8338	465.1487	0.0673
Total.Intracranial.Vol	0.5871	0.0376	15.5938	465.0022	0.0000
Childhood.Stress	-0.0586	0.0322	-1.8219	464.1777	0.0691
Pos.Parenting.Youth.Report	-0.0027	0.0329	-0.0814	464.2707	0.9351

p values calculated using Satterthwaite d.f.

Random Effects		
Group	Parameter	Std. Dev.
Site	(Intercept)	0.3573
Residual		0.6861

Grouping Variables		
Group	# groups	ICC
Site	4	0.2133

Model Output. This table depicts mixed effects linear regression model output, including both fixed effects and random effects. Fixed effects include standardized regression coefficients (Est.), standard errors (S.E.), t-values (t val.), and p-values (p-val). Random effects include the random effect of Imaging Site and the number of grouping variables (4 imaging sites). Model output also depicts overall model fit statistics and number of observations.

Childhood Stress and Caregiver-Report Parenting on Total Hippocampal Volume

Observations	474
Dependent variable	TotalHippocampalVolume
Type	Mixed effects linear regression

AIC	1046.3439
BIC	1087.9559
Pseudo-R^2 (fixed effects)	0.4150
Pseudo-R^2 (total)	0.5393

Fixed Effects					
	Est.	S.E.	t val.	d.f.	p
(Intercept)	-0.1401	0.1907	-0.7346	3.4241	0.5097
Scan.Quality	0.0863	0.0389	2.2163	457.9293	0.0272
SexMale	0.1253	0.0770	1.6279	463.4864	0.1042
Age	0.0748	0.0329	2.2759	463.3851	0.0233
Barratt.Edu.Occ	0.0658	0.0343	1.9192	464.1915	0.0556
Total.Intracranial.Vol	0.5864	0.0375	15.6355	464.0663	0.0000
Childhood.Stress	-0.0594	0.0320	-1.8547	463.1759	0.0643
Pos.Parenting.Caregiver.Report	0.0387	0.0330	1.1740	463.8670	0.2410

p values calculated using Satterthwaite d.f.

Random Effects		
Group	Parameter	Std. Dev.
Site	(Intercept)	0.3562
Residual		0.6855

Grouping Variables		
Group	# groups	ICC
Site	4	0.2126

Model Output. This table depicts mixed effects linear regression model output, including both fixed effects and random effects. Fixed effects include standardized regression coefficients (Est.), standard errors (S.E.), t-values (t val.), and p-values (p-val). Random effects include the random effect of Imaging Site and the number of grouping variables (4 imaging sites). Model output also depicts overall model fit statistics and number of observations.

Childhood Stress and Youth-Report Parenting on Total Amygdala

Observations	475
Dependent variable	TotalAmygdalaVolume
Type	Mixed effects linear regression

AIC	1089.6543
BIC	1131.2874
Pseudo-R^2 (fixed effects)	0.3785
Pseudo-R^2 (total)	0.4921

Fixed Effects					
	Est.	S.E.	t val.	d.f.	p
(Intercept)	-0.3132	0.1838	-1.7045	3.5716	0.1720
Scan.Quality	0.1082	0.0406	2.6643	455.0092	0.0080
SexMale	0.3105	0.0802	3.8723	464.7161	0.0001
Age	0.0797	0.0344	2.3185	464.6865	0.0209
Barratt.Edu.Occ	0.0103	0.0358	0.2874	465.3594	0.7740
Total.Intracranial.Vol	0.4951	0.0393	12.5885	465.1911	0.0000
Childhood.Stress	-0.0317	0.0336	-0.9437	464.2779	0.3458
Pos.Parenting.Youth.Report	-0.0433	0.0344	-1.2596	464.3883	0.2084

p values calculated using Satterthwaite d.f.

Random Effects		
Group	Parameter	Std. Dev.
Site	(Intercept)	0.3391
Residual		0.7168

Grouping Variables		
Group	# groups	ICC
Site	4	0.1829

Model Output. This table depicts mixed effects linear regression model output, including both fixed effects and random effects. Fixed effects include standardized regression coefficients (Est.), standard errors (S.E.), t-values (t val.), and p-values (p-val). Random effects include the random effect of Imaging Site and the number of grouping variables (4 imaging sites). Model output also depicts overall model fit statistics and number of observations.

Childhood Stress and Caregiver-Report Parenting on Total Amygdala

Observations	474
Dependent variable	TotalAmygdalaVolume
Type	Mixed effects linear regression

AIC	1089.8100
BIC	1131.4221
Pseudo-R^2 (fixed effects)	0.3760
Pseudo-R^2 (total)	0.4910

Fixed Effects					
	Est.	S.E.	t val.	d.f.	p
(Intercept)	-0.3106	0.1849	-1.6793	3.5721	0.1769
Scan.Quality	0.1120	0.0407	2.7486	452.9536	0.0062
SexMale	0.3079	0.0807	3.8147	463.6223	0.0002
Age	0.0848	0.0345	2.4593	463.5061	0.0143
Barratt.Edu.Occ	0.0084	0.0359	0.2325	464.3867	0.8163
Total.Intracranial.Vol	0.4997	0.0393	12.7121	464.2461	0.0000
Childhood.Stress	-0.0353	0.0336	-1.0501	463.2672	0.2942
Pos.Parenting.Caregiver.Report	-0.0167	0.0346	-0.4836	464.0223	0.6289

p values calculated using Satterthwaite d.f.

Random Effects		
Group	Parameter	Std. Dev.
Site	(Intercept)	0.3415
Residual		0.7186

Grouping Variables		
Group	# groups	ICC
Site	4	0.1842

Model Output. This table depicts mixed effects linear regression model output, including both fixed effects and random effects. Fixed effects include standardized regression coefficients (Est.), standard errors (S.E.), t-values (t val.), and p-values (p-val). Random effects include the random effect of Imaging Site and the number of grouping variables (4 imaging sites). Model output also depicts overall model fit statistics and number of observations.

Examining Associations Between Youth Behavioral Problems, Stress, Parenting, and an Interaction of Stress X Parenting

Caregiver-Report of Youth Behavioral Problems

In our main manuscript, we detail results of a model considering associations between youth behavioral problems (from the Youth-Self Report [YSR] Total Score) in relation to childhood stress, parenting (youth-report), and the interaction of stress and parenting. This was due to our interest in centering youth perspectives in our research. The results of this model suggested a potential buffering effect of positive parenting within the association between stress and behavioral problems. Put another way. there is no relation between stress and behavioral problems, for children exposed to the highest levels of positive parenting.

Associations between Youth-Report Stress and Youth-Report Parenting on SDQ Total Score

The results of this model suggest a significant interaction of childhood stress and youth-reported positive parenting on caregiver-reported youth problems (i.e., SDQ total difficulties score). This replication of our above result (i.e., with the YSR as an outcome variable instead of the SDQ) underscores that there is an interaction between childhood stress and youth-reported positive parenting on youth behavioral problems regardless of whether the youth or caregiver is the informant on youth behavioral problems.

Observations	400 (82 missing obs. deleted)
Dependent variable	Strengths.Difficulties.Total
Type	OLS linear regression

F(9,390)	3.3692
R^2	0.0721
Adj. R^2	0.0507

	Est.	S.E.	t val.	p
(Intercept)	-0.1419	0.0972	-1.4608	0.1449
SexMale	0.1439	0.1020	1.4108	0.1591
Age	-0.1173	0.0616	-1.9041	0.0576
Barratt.Edu.Occ	-0.1959	0.0539	-3.6340	0.0003
SiteCUNY	0.4226	0.2929	1.4429	0.1499
SiteRU	0.1292	0.1079	1.1977	0.2318
SiteSI	0.1930	0.1589	1.2150	0.2251
Childhood.Stress	0.0308	0.0517	0.5960	0.5515
Pos.Parenting.Youth.Report	0.0136	0.0520	0.2624	0.7931
Childhood.Stress:Pos.Parenting.Youth.Report	-0.0962	0.0479	-2.0100	0.0451

Standard errors: OLS

Model Output. This table depicts linear regression model output, including standardized regression coefficients (Est.), standard errors (S.E.), t-values (t val.), and p-values (p-val). Model output also depicts overall model fit statistics and number of observations.

Associations between Youth-Report Stress and Caregiver-Report Parenting on SDQ Total Score

This next model investigated the interaction of childhood stress and caregiver-reported positive parenting on caregiver-reported youth problems (i.e., SDQ total difficulties score). Consistent with previous results, there is no significant interaction between childhood stress and caregiver-reported positive parenting on behavioral problems.

Observations	399 (83 missing obs. deleted)
Dependent variable	Strengths.Difficulties.Total
Type	OLS linear regression

F(9,389)	3.0415
R^2	0.0657
Adj. R^2	0.0441

	Est.	S.E.	t val.	p
(Intercept)	-0.1272	0.0978	-1.3010	0.1940
SexMale	0.1371	0.1029	1.3324	0.1835
Age	-0.1158	0.0612	-1.8941	0.0590
Barratt.Edu.Occ	-0.1944	0.0541	-3.5921	0.0004
SiteCUNY	0.3339	0.2939	1.1364	0.2565
SiteRU	0.1070	0.1080	0.9911	0.3223
SiteSI	0.1833	0.1593	1.1505	0.2506
Childhood.Stress	0.0255	0.0514	0.4956	0.6205
Pos.Parenting.Caregiver.Report	0.0554	0.0506	1.0940	0.2746
Childhood.Stress:Pos.Parenting.Caregiver.Report	-0.0349	0.0502	-0.6959	0.4869

Standard errors: OLS

Model Output. This table depicts linear regression model output, including standardized regression coefficients (Est.), standard errors (S.E.), t-values (t val.), and p-values (p-val). Model output also depicts overall model fit statistics and number of observations.

Socioeconomic Status (SES) Variables

Most studies to date exploring stress and parenting on youth outcomes have operationalized stress as socioeconomic disadvantage, while less research has focused on exposure to stressful and negative life events. In order to test the unique contribution of actual experience of stress on youth behavioral problems and corticolimbic structure and the moderating effect of positive parenting, we consider below various operationalizations of SES as covariates in our models and correlations with study variables.

Correlations between Stress and Multiple Operationalizations of SES

We considered correlations between Childhood Stress (youth-reported Negative Life Events) and four different operationalizations of socioeconomic status, including a) a categorical variable representing parent employment status (Parental.Employment), b) a log-transformed income variable (Income), c) a variable representing the highest parental education achievement and occupational status from the Barratt Simplified Measure of Social Status (BSMSS) (Barratt.Edu.Occ), and d) the Barratt measure total "social status" score (Barratt.Total).

As reflected in the results below, the correlations between stress and SES variables are quite low (ranging from r = -0.0085 to -0.0314) and non-significant at the $p < 0.05$ level.

The correlation between FSQ categorical parental employment and stress is r = -0.031, 95% CI [-0.1278, 0.0656], t = -0.634, df = 408, p-value = 0.5262.

The correlation between FSQ income variable and stress is r = -0.033, 95% CI [-0.1354, 0.0708], t = -0.619, df = 359, p-value = 0.5365.

The correlation between Barratt Z score mean parental education/occupation and stress is r = -0.0085, 95% CI [-0.0984, 0.0815], t = -0.185, df = 473, p-value = 0.853.

The correlation between Barratt total score and stress is r = -0.003, 95% CI [-0.0929, 0.0866], t = -0.069, df = 475, p-value = 0.9449.

Correlations between Parenting and Multiple Operationalizations of SES

We considered correlations between both a) youth-report Positive Parenting and b) caregiver-report Positive Parenting and each of the 4 SES variables described below.

All but one of these 8 correlations were very low and non-significant at the $p < 0.05$ level, with the exception of the correlation between caregiver-report Positive Parenting and Income (r = -0.1182, p = 0.02486).

The correlations between FSQ categorical parental employment and child report parenting is r = 0.0503, 95% CI [-0.0467, 0.1465], t = 1.018, df = 408, p-value = 0.3091.

The correlation between FSQ categorical parental employment and caregiver-report parenting is r = -0.022, 95% CI [-0.1139, 0.0749], t = -0.4495, df = 407, p-value = 0.6533.

The correlation between FSQ income variable and child-report parenting is r = -0.054, 95% CI [-0.1563, 0.0496], t = -1.023, df = 359, p-value = 0.3068.

The correlation between FSQ income variable and caregiver-report parenting is r = -0.118, 95% CI [-0.2189, -0.0151], t = -2.253, df = 358; p-value = 0.02486.

The correlations between Barratt Z score mean parental education/occupation and child-report parenting is r = -0.011, 95% CI [-0.1012, 0.0788], t = -0.246, df = 473, p-value = 0.806.

The correlations between Barratt Z score mean parental education/occupation and caregiver-report parenting is r = - 0.074, 95% CI [-0.1631, 0.0161], t = -1.6142, df = 472, p-value = 0.1071.

The correlation between Barratt total and child-report parenting is r = -0.027, 95% CI [-0.1163, 0.0631], t = -0.585, df = 475, p-value = 0.5591.

The correlation between Barratt total and child-report parenting is r = -0.082, 95% CI [-0.1708, 0.0077], t = -1.796, df = 474, p-value = 0.0732.

Sensitivity Analysis with Socioeconomic Status (SES) as an Additional Variable in Main Models

Childhood Stress, Youth-Report Parenting, and SES on Youth-Report Behavioral Problems

We ran models calculating the association between Childhood stress and youth-report parenting on youth-report behavioral problems using 4 different operationalizations of socioeconomic status: a) a categorical variable representing parent employment status (Parental.Employment), b) a log-transformed income variable (Income), c) a variable representing the highest parental education achievement and occupational status from the Barratt Simplified Measure of Social Status (BSMSS) (Barratt.Edu.Occ), and d) the Barratt measure total "social status" score (Barratt.Total).

Analyses using the parent employment status variable (Parental.Employment) led to a reduction in sample size from n = 410 to n = 358. Analyses using the income variable (income) led to a reduction in sample size from n = 410 to n = 313.

Analyses using the variables from the Barratt measure (ie., a variable representing the highest level of parental education attainment and occupation status [Barratt.Edu.Occ] and a variable representing overall social status [Barratt.Total]) preserved our sample size the most, with a reduction in sample from n = 410 to n = 404. In order to preserve as large a sample size as possible, we used the Barratt.Edu.Occ variable as a covariate in all our main analyses.

Stress * Youth-Report Parenting on Youth Behavioral Problems with Categorical Parental Employment as SES Variable

Observations	358 (124 missing obs. deleted)
Dependent variable	Youth.Self.Report.Total
Type	OLS linear regression

F(9,348)	3.4565
R^2	0.0821
Adj. R^2	0.0583

	Est.	S.E.	t val.	p
(Intercept)	0.1797	0.1689	1.0636	0.2882
SexMale	-0.0462	0.1042	-0.4433	0.6578
Age	0.0998	0.0628	1.5895	0.1128
SiteCUNY	-0.0563	0.2836	-0.1985	0.8427
SiteRU	-0.0022	0.1052	-0.0213	0.9830
SiteSI	0.1331	0.2749	0.4842	0.6286
Parental.Employment	-0.1326	0.0870	-1.5243	0.1283
Childhood.Stress	0.1320	0.0525	2.5146	0.0124
Pos.Parenting.Youth.Report	-0.2023	0.0545	-3.7149	0.0002
Childhood.Stress:Pos.Parenting.Youth.Report	-0.0659	0.0501	-1.3159	0.1891

Standard errors: OLS

Model Output. This table depicts linear regression model output, including standardized regression coefficients (Est.), standard errors (S.E.), t-values (t val.), and p-values (p-val). Model output also depicts overall model fit statistics and number of observations.

Stress * Youth-Report Parenting on Youth Behavioral Problems with Income as SES Variable

Observations	313 (169 missing obs. deleted)
Dependent variable	Youth.Self.Report.Total
Type	OLS linear regression

F(9,303)	5.2961
R^2	0.1359
Adj. R^2	0.1103

	Est.	S.E.	t val.	p
(Intercept)	-0.0534	0.1049	-0.5094	0.6109
SexMale	-0.0045	0.1140	-0.0398	0.9683
Age	0.1327	0.0693	1.9165	0.0562
SiteCUNY	0.0108	0.2915	0.0370	0.9705
SiteRU	0.0062	0.1137	0.0547	0.9564
SiteSI	0.1571	0.3345	0.4697	0.6389
Income	-0.1567	0.0567	-2.7648	0.0060
Childhood.Stress	0.1846	0.0572	3.2285	0.0014
Pos.Parenting.Youth.Report	-0.2659	0.0589	-4.5120	0.0000
Childhood.Stress:Pos.Parenting.Youth.Report	-0.0857	0.0530	-1.6161	0.1071

Standard errors: OLS

Model Output. This table depicts linear regression model output, including standardized regression coefficients (Est.), standard errors (S.E.), t-values (t val.), and p-values (p-val). Model output also depicts overall model fit statistics and number of observations.

Stress * Youth-Report Parenting on Youth Behavioral Problems with Highest Parental Education & Occupational Status as SES Variable

Observations	404 (78 missing obs. deleted)
Dependent variable	Youth.Self.Report.Total
Type	OLS linear regression

F(9,394)	5.5898
R^2	0.1132
Adj. R^2	0.0930

	Est.	S.E.	t val.	p
(Intercept)	-0.0095	0.0944	-0.1010	0.9196
SexMale	-0.0892	0.0991	-0.8998	0.3688
Age	0.1089	0.0600	1.8148	0.0703
SiteCUNY	0.0057	0.2861	0.0200	0.9840
SiteRU	0.0277	0.1053	0.2636	0.7923
SiteSI	0.0994	0.1520	0.6539	0.5135
Barratt.Edu.Occ	-0.1144	0.0525	-2.1792	0.0299
Childhood.Stress	0.1371	0.0503	2.7248	0.0067
Pos.Parenting.Youth.Report	-0.2482	0.0507	-4.8966	0.0000
Childhood.Stress:Pos.Parenting.Youth.Report	-0.0963	0.0467	-2.0607	0.0400

Standard errors: OLS

Model Output. This table depicts linear regression model output, including standardized regression coefficients (Est.), standard errors (S.E.), t-values (t val.), and p-values (p-val). Model output also depicts overall model fit statistics and number of observations.

Stress * Youth-Report Parenting on Youth Behavioral Problems with Barratt Measure Social Status as SES Variable

Observations	406 (76 missing obs. deleted)
Dependent variable	Youth.Self.Report.Total
Type	OLS linear regression

F(9,396)	5.6091
R^2	0.1131
Adj. R^2	0.0929

	Est.	S.E.	t val.	p
(Intercept)	-0.0067	0.0945	-0.0708	0.9436
SexMale	-0.0724	0.0989	-0.7323	0.4644
Age	0.1150	0.0601	1.9129	0.0565
SiteCUNY	0.0134	0.2871	0.0468	0.9627
SiteRU	0.0169	0.1047	0.1617	0.8716
SiteSI	0.0769	0.1527	0.5033	0.6150
Barratt.Total	-0.1131	0.0491	-2.3025	0.0218
Childhood.Stress	0.1418	0.0503	2.8178	0.0051
Pos.Parenting.Youth.Report	-0.2476	0.0505	-4.9076	0.0000
Childhood.Stress:Pos.Parenting.Youth.Report	-0.0885	0.0467	-1.8952	0.0588

Standard errors: OLS

Model Output. This table depicts linear regression model output, including standardized regression coefficients (Est.), standard errors (S.E.), t-values (t val.), and p-values (p-val). Model output also depicts overall model fit statistics and number of observations.

Childhood Stress, Youth-Report Parenting, and SES on Total Hippocampal Volume

Given the varying sample sizes for the different operationalizations, it is difficult to draw across-the-board conculsions regaring the role of socioeconomic status within our models exploring the interactive effect between childhood stress and positive parenting on youth behavioral problems and total hippocampal volumes. However, in the analyses that preserve the largest sample sizes (i.e., in considering SES variables from the Barratt measure) it appears that socioeconomic status does not explain the significant associations between chilhood stress, youth-reported positive parenting, and smaller hippocampal volumes.

Stress * Youth-Report Parenting on Total Hippocampal Volume with Categorical Parental Employment as SES Variable

Observations	410
Dependent variable	TotalHippocampalVolume
Type	Mixed effects linear regression

AIC	890.6386
BIC	934.8163
Pseudo-R^2 (fixed effects)	0.4798
Pseudo-R^2 (total)	0.5867

Fixed Effects					
	Est.	S.E.	t val.	d.f.	p
(Intercept)	-0.1681	0.2032	-0.8271	4.4824	0.4500
Scan.Quality	0.0339	0.0389	0.8716	396.0640	0.3840
SexMale	0.0268	0.0824	0.3250	398.7149	0.7453
Age	0.2704	0.0362	7.4723	398.3184	0.0000
Total.Gray.Vol	0.6870	0.0413	16.6189	399.4189	0.0000
Parental.Employment	0.0529	0.0565	0.9352	398.5218	0.3502
Childhood.Stress	-0.0798	0.0341	-2.3362	397.7877	0.0200
Pos.Parenting.Youth.Report	-0.0191	0.0349	-0.5476	398.1240	0.5842
Childhood.Stress:Pos.Parenting.Youth.Report	0.0825	0.0324	2.5466	399.4390	0.0113

p values calculated using Satterthwaite d.f.

Random Effects		
Group	Parameter	Std. Dev.
Site	(Intercept)	0.3380
Residual		0.6645

Grouping Variables		
Group	# groups	ICC
Site	4	0.2056

Model Output. This table depicts mixed effects linear regression model output, including both fixed effects and random effects. Fixed effects include standardized regression coefficients (Est.), standard errors (S.E.), t-values (t val.), and p-values (p-val). Random effects include the random effect of Imaging Site and the number of grouping variables (4 imaging sites). Model output also depicts overall model fit statistics and number of observations.

Stress * Youth-Report Parenting on Total Hippocampal Volume with Income as SES Variable

Observations	361
Dependent variable	TotalHippocampalVolume
Type	Mixed effects linear regression

AIC	781.8405
BIC	824.6181
Pseudo-R^2 (fixed effects)	0.4973
Pseudo-R^2 (total)	0.5701

Fixed Effects					
	Est.	S.E.	t val.	d.f.	p
(Intercept)	-0.0812	0.1569	-0.5174	2.6717	0.6446
Scan.Quality	0.0204	0.0402	0.5085	338.3092	0.6114
SexMale	0.0573	0.0860	0.6662	349.2381	0.5057
Age	0.2647	0.0387	6.8493	349.5675	0.0000
Total.Gray.Vol	0.6639	0.0430	15.4293	350.8558	0.0000
Income	0.0684	0.0356	1.9205	350.5271	0.0556
Childhood.Stress	-0.0650	0.0360	-1.8054	348.2520	0.0719
Pos.Parenting.Youth.Report	-0.0057	0.0360	-0.1572	348.2982	0.8752
Childhood.Stress:Pos.Parenting.Youth.Report	0.0644	0.0336	1.9161	350.4866	0.0562

p values calculated using Satterthwaite d.f.

Random Effects		
Group	Parameter	Std. Dev.
Site	(Intercept)	0.2703
Residual		0.6568

Grouping Variables		
Group	# groups	ICC
Site	4	0.1448

Model Output. This table depicts mixed effects linear regression model output, including both fixed effects and random effects. Fixed effects include standardized regression coefficients (Est.), standard errors (S.E.), t-values (t val.), and p-values (p-val). Random effects include the random effect of Imaging Site and the number of grouping variables (4 imaging sites). Model output also depicts overall model fit statistics and number of observations.

Stress * Youth-Report Parenting on Total Hippocampal Volume with Highest Parental Education & Occupational Status as SES Variable

Observations	475
Dependent variable	TotalHippocampalVolume
Type	Mixed effects linear regression

AIC	1014.2807
BIC	1060.0772
Pseudo-R^2 (fixed effects)	0.4600
Pseudo-R^2 (total)	0.5783

Fixed Effects					
	Est.	S.E.	t val.	d.f.	p
(Intercept)	-0.0939	0.1858	-0.5054	3.4157	0.6441
Scan.Quality	0.0272	0.0375	0.7254	459.3030	0.4686
SexMale	0.0360	0.0748	0.4812	463.6720	0.6306
Age	0.2714	0.0323	8.3981	463.5982	0.0000
Total.Gray.Vol	0.6609	0.0377	17.5299	464.3849	0.0000
Barratt.Edu.Occ	0.0417	0.0330	1.2665	463.9661	0.2060
Childhood.Stress	-0.0901	0.0313	-2.8746	463.1411	0.0042
Pos.Parenting.Youth.Report	0.0070	0.0317	0.2211	463.2263	0.8251
Childhood.Stress:Pos.Parenting.Youth.Report	0.0653	0.0293	2.2284	463.9234	0.0263

p values calculated using Satterthwaite d.f.

Random Effects		
Group	Parameter	Std. Dev.
Site	(Intercept)	0.3476
Residual		0.6565

Grouping Variables		
Group	# groups	ICC
Site	4	0.2190

Model Output. This table depicts mixed effects linear regression model output, including both fixed effects and random effects. Fixed effects include standardized regression coefficients (Est.), standard errors (S.E.), t-values (t val.), and p-values (p-val). Random effects include the random effect of Imaging Site and the number of grouping variables (4 imaging sites). Model output also depicts overall model fit statistics and number of observations.

Stress * Youth-Report Parenting on Total Hippocampal Volume with Barratt Measure Social Status as SES Variable

Observations	477
Dependent variable	TotalHippocampalVolume
Type	Mixed effects linear regression

AIC	1017.3524
BIC	1063.1951
Pseudo-R^2 (fixed effects)	0.4615
Pseudo-R^2 (total)	0.5791

Fixed Effects					
	Est.	S.E.	t val.	d.f.	p
(Intercept)	-0.0954	0.1854	-0.5148	3.4160	0.6382
Scan.Quality	0.0265	0.0373	0.7112	460.8663	0.4773
SexMale	0.0331	0.0747	0.4431	465.7354	0.6579
Age	0.2720	0.0323	8.4311	465.6505	0.0000
Total.Gray.Vol	0.6619	0.0376	17.5842	466.3551	0.0000
Barratt.Total	0.0292	0.0310	0.9438	466.7632	0.3457
Childhood.Stress	-0.0904	0.0312	-2.8933	465.1395	0.0040
Pos.Parenting.Youth.Report	0.0062	0.0314	0.1987	465.2188	0.8426
Childhood.Stress:Pos.Parenting.Youth.Report	0.0661	0.0292	2.2640	465.8238	0.0240

p values calculated using Satterthwaite d.f.

Random Effects		
Group	Parameter	Std. Dev.
Site	(Intercept)	0.3466
Residual		0.6557

Grouping Variables		
Group	# groups	ICC
Site	4	0.2184

Model Output. This table depicts mixed effects linear regression model output, including both fixed effects and random effects. Fixed effects include standardized regression coefficients (Est.), standard errors (S.E.), t-values (t val.), and p-values (p-val). Random effects include the random effect of Imaging Site and the number of grouping variables (4 imaging sites). Model output also depicts overall model fit statistics and number of observations.

Cognitive Ability as a Covariate in Main Analyses

Correlations between stress and IQ

We used the WISC Full Scale Intelligence Quotient (FSIQ) score as an index of cognitive ability. We considered the correlation between FSIQ and stress, finding that the two constructs were not correlated (r = 0.0117, 95% CI [-0.0890, 0.11217], t = 0.22753, df = 378, p-value = 0.8201).

IQ as a covariate in main analyses

Childhood Stress and Youth-Report Parenting on Youth-Report Behavioral Problems with IQ

The inclusion of WISC IQ score leads to an over 20% reduction in our sample size (from n = 410 to n = 320). We therefore chose not to include IQ as a covariate in our main analyses.

Observations	320 (162 missing obs. deleted)
Dependent variable	Youth.Self.Report.Total
Type	OLS linear regression

F(9,310)	2.0502
R^2	0.0562
Adj. R^2	0.0288

	Est.	S.E.	t val.	p
(Intercept)	-0.0875	0.1010	-0.8669	0.3867
SexMale	-0.0534	0.1108	-0.4818	0.6303
Age	0.0918	0.0817	1.1241	0.2618
SiteCUNY	0.1275	0.3090	0.4126	0.6802
SiteRU	0.0958	0.1135	0.8434	0.3997
SiteSI	0.3604	0.2188	1.6475	0.1005
WISC.FSIQ	0.0062	0.0538	0.1144	0.9090
Childhood.Stress	0.0759	0.0564	1.3458	0.1794
Pos.Parenting.Youth.Report	-0.1773	0.0596	-2.9741	0.0032
Childhood.Stress:Pos.Parenting.Youth.Report	-0.0384	0.0555	-0.6911	0.4900

Standard errors: OLS

Model Output. This table depicts linear regression model output, including standardized regression coefficients (Est.), standard errors (S.E.), t-values (t val.), and p-values (p-val). Model output also depicts overall model fit statistics and number of observations.

Childhood Stress and Youth-Report Parenting with IQ on Total Hippocampal Volume

Observations	380
Dependent variable	TotalHippocampalVolume
Type	Mixed effects linear regression

AIC	859.6452
BIC	902.9871
Pseudo-R^2 (fixed effects)	0.4454
Pseudo-R^2 (total)	0.5589

Fixed Effects					
	Est.	S.E.	t val.	d.f.	p
(Intercept)	-0.1834	0.1920	-0.9549	3.4238	0.4021
Scan.Quality	0.0817	0.0426	1.9191	363.4225	0.0558
SexMale	0.1491	0.0881	1.6922	368.2501	0.0914
Age	0.0695	0.0446	1.5575	368.5390	0.1202
Total.Intracranial.Vol	0.6129	0.0431	14.2216	368.9984	0.0000
WISC.FSIQ	0.0783	0.0376	2.0836	369.2794	0.0379
Childhood.Stress	-0.0523	0.0378	-1.3817	368.3457	0.1679
Pos.Parenting.Youth.Report	0.0235	0.0386	0.6076	368.1475	0.5438
Childhood.Stress:Pos.Parenting.Youth.Report	0.0858	0.0369	2.3230	368.9945	0.0207

p values calculated using Satterthwaite d.f.

Random Effects		
Group	Parameter	Std. Dev.
Site	(Intercept)	0.3507
Residual		0.6915

Grouping Variables		
Group	# groups	ICC
Site	4	0.2046

Model Output. This table depicts mixed effects linear regression model output, including both fixed effects and random effects. Fixed effects include standardized regression coefficients (Est.), standard errors (S.E.), t-values (t val.), and p-values (p-val). Random effects include the random effect of Imaging Site and the number of grouping variables (4 imaging sites). Model output also depicts overall model fit statistics and number of observations.

Childhood Stress and Caregiver-Report Parenting with IQ on Total Hippocampal Volume

Observations	379
Dependent variable	TotalHippocampalVolume
Type	Mixed effects linear regression

AIC	860.7609
BIC	904.0738
Pseudo-R^2 (fixed effects)	0.4408
Pseudo-R^2 (total)	0.5569

Fixed Effects					
	Est.	S.E.	t val.	d.f.	p
(Intercept)	-0.1809	0.1944	-0.9303	3.4149	0.4132
Scan.Quality	0.0770	0.0428	1.7985	362.1152	0.0729
SexMale	0.1581	0.0885	1.7862	367.2677	0.0749
Age	0.0813	0.0453	1.7948	367.3972	0.0735
Total.Intracranial.Vol	0.6035	0.0432	13.9820	367.8605	0.0000
WISC.FSIQ	0.0814	0.0374	2.1750	368.1089	0.0303
Childhood.Stress	-0.0342	0.0371	-0.9215	367.6252	0.3574
Pos.Parenting.Caregiver.Report	0.0487	0.0378	1.2883	368.3474	0.1985
Childhood.Stress:Pos.Parenting.Caregiver.Report	0.0393	0.0360	1.0902	367.2269	0.2764

p values calculated using Satterthwaite d.f.

Random Effects		
Group	Parameter	Std. Dev.
Site	(Intercept)	0.3554
Residual		0.6944

Grouping Variables		
Group	# groups	ICC
Site	4	0.2076

Model Output. This table depicts mixed effects linear regression model output, including both fixed effects and random effects. Fixed effects include standardized regression coefficients (Est.), standard errors (S.E.), t-values (t val.), and p-values (p-val). Random effects include the random effect of Imaging Site and the number of grouping variables (4 imaging sites). Model output also depicts overall model fit statistics and number of observations.

Sensitivity Analysis with Psychopathology Diagnosis as an Additional Variable in Main Models

Given past research noting connections between psychopathology and hippocampal neurobiology, we constructed additional models that included a measure of psychopathology in our models examining stress, parenting, and the interaction of stress and parenting. We used a binary indicator of the presence (or absence) of psychopathology derived from the Kiddie Schedule for Affective Disorders and Schizophrenia (KSADS) Diagnostic Interview. This variable was originally coded as 1= "No", 2= "Yes", 3= "dropped out of study before diagnosis was given". We recoded this variable to exclude datapoints with a value of 3 for the purposes of this analysis. We named this binary diagnosis variable *Any.Psychopathology* as presented in the following model.

Upon selecting datapoints for which the *Any.Psychopathology* variable was available, there is a *large* drop in sample size for these analyses (n=226 vs. n=400-480 in other models). We first replicated our main analysis of interest among this smaller sample (i.e., childhood stress and youth-reported positive parenting predicting total hippocampal volumes) and found that the main effect of childhood stress and the interaction between childhood stress and youth-reported positive parenting is no longer significant in this smaller sample.

Youth-Report Parenting and Stress on Total Hippocampal Volume among Subsample with Psychopathology Data (LMEM)

Observations	226
Dependent variable	TotalHippocampalVolume
Type	Mixed effects linear regression

AIC	3522.2624
BIC	3556.4677
Pseudo-R^2 (fixed effects)	0.3820
Pseudo-R^2 (total)	0.5759

Fixed Effects					
	Est.	S.E.	t val.	d.f.	p
(Intercept)	1559.8880	3281.6706	0.4753	167.6809	0.6352
Scan.Quality	2007.1044	3707.2656	0.5414	168.7568	0.5889
SexMale	34.5019	99.7677	0.3458	216.2049	0.7298
Age	38.6942	16.8857	2.2915	216.7559	0.0229
Total.Intracranial.Vol	0.0032	0.0003	11.5970	216.2199	0.0000
Childhood.Stress	-306.1938	195.3132	-1.5677	216.0441	0.1184
Pos.Parenting.Youth.Report	-13.0135	25.0842	-0.5188	216.0331	0.6044
Childhood.Stress:Pos.Parenting.Youth.Report	10.4709	7.9549	1.3163	216.0612	0.1895

p values calculated using Satterthwaite d.f.

Random Effects		
Group	Parameter	Std. Dev.
Site	(Intercept)	426.7487
Residual		630.9738

Grouping Variables		
Group	# groups	ICC
Site	3	0.3139

Model Output. This table depicts mixed effects linear regression model output, including both fixed effects and random effects. Fixed effects include standardized regression coefficients (Est.), standard errors (S.E.), t-values (t val.), and p-values (p-val). Random effects include the random effect of Imaging Site and the number of grouping variables (4 imaging sites). Model output also depicts overall model fit statistics and number of observations.

Youth-Report Parenting, Stress, and Psychopathology on Total Hippocampal Volume (LMEM)

The binary diagnosis variable (i.e., *Any.Psychopathology*) was added to our main model predicting total hippocampal volume from an interaction between stress and youth-reported positive parenting, along with our main covariate set. The variable *Any.Psychopathology* was not significantly associated with total hippocampal volumes in this sample.

Observations	226
Dependent variable	TotalHippocampalVolume
Type	Mixed effects linear regression

AIC	3512.8009
BIC	3550.4268
Pseudo-R^2 (fixed effects)	0.3823
Pseudo-R^2 (total)	0.5738

Fixed Effects					
	Est.	S.E.	t val.	d.f.	p
(Intercept)	1544.4179	3287.3407	0.4698	165.7482	0.6391
Scan.Quality	2040.0935	3714.3295	0.5492	166.3217	0.5836
SexMale	32.4425	100.3607	0.3233	215.1647	0.7468
Age	37.6754	17.4197	2.1628	215.8401	0.0317
Total.Intracranial.Vol	0.0032	0.0003	11.4850	215.2465	0.0000
Childhood.Stress	-310.4362	196.5039	-1.5798	215.0688	0.1156
Pos.Parenting.Youth.Report	-13.5456	25.2344	-0.5368	215.0425	0.5920
Any.PsychopathologyYes Dx	29.2002	119.4710	0.2444	215.5920	0.8071
Childhood.Stress:Pos.Parenting.Youth.Report	10.6547	8.0073	1.3306	215.0919	0.1847

p values calculated using Satterthwaite d.f.

Random Effects		
Group	Parameter	Std. Dev.
Site	(Intercept)	423.9398
Residual		632.3876

Grouping Variables		
Group	# groups	ICC
Site	3	0.3101

Model Output. This table depicts mixed effects linear regression model output, including both fixed effects and random effects. Fixed effects include standardized regression coefficients (Est.), standard errors (S.E.), t-values (t val.), and p-values (p-val). Random effects include the random effect of Imaging Site and the number of grouping variables (4 imaging sites). Model output also depicts overall model fit statistics and number of observations.

Probing Questions of Interest Using Generalized Additive Mixed Models (GAMMs) to Account for Potential Non-Linear Age Effects

Youth-Report Parenting and Stress on Total Hippocampal Volume (GAMM)

In the main manuscript, we used linear mixed effects models to examine relations of interest. However, there may be non-linear effects of age on brain volume. To model these potential patterns of change, we fit Generalized Additive Mixed Models (GAMMs) with package 'gamm4', "Generalized Additive Mixed Models using 'mgcv' and 'lme4' " (v 0.2-6, Woods & Scheipl, 2020). GAMMs allow for modeling of non-linear data by incorporating smooth functions, or splines, and accounting for random effects (e.g., study site) using mixed effects models. This is particularly critical to flexibly capture linear or nonlinear age effects and is in keeping with recent cutting edge work in developmental cognitive neuroscience (Larsen et al., 2020).

For each brain region of interest (i.e., total hippocampal volume, total amygdala volume), we first fit a GAMM with a random effect of site, a smooth (non-linear) term for age, and main effects of sex, estimated total intracranial volume ($Total.Intracranial.Vol$), negative life events, youth-reported positive parenting, and an interaction between negative life events and youth-reported positive parenting. This was motivated by our interest in youth perceptions of parenting behaviors. Notably, we also fit a second set of GAMMs for each brain region of interest again including a random effect of site, a smooth term for age, and main effects of sex, total intracranial volume, and Youth-Reported negative life events, this time including caregiver-reported positive parenting and an interaction between youth-reported negative life events and caregiver-reported positive parenting. This was to examine the importance of youth (as opposed to caregiver) reports of parenting. The results of this GAMM are consistent with the LMEM reported in the manuscript; namely, that there is a significant interaction between stress and youth-reported positive parenting on total hippocampal volume.

Youth-Report Parenting and Stress on Total Hippocampal Volume GAMM Model Output

Model Title: Youth-Report Parenting and Stress on Total Hippocampal Volume (GAMM)

Observations: 482

Linear Mixed Effects Model

Fixed Effects

	Estimate	Std. Error	t-value
Intercept	-0.137220	0.192294	-0.714
Scan Quality	0.086345	0.038522	2.241
Sex (Male)	0.102049	0.075720	1.348
Total Intracranial Volume	0.596887	0.037139	16.072
Childhood Stress	-0.070518	0.032226	-2.177
Positive Parenting Youth Report	0.005024	0.032618	0.154
Childhood Stress * Pos. Parenting Youth Report	0.070110	0.030193	2.322
Age (smoothing term)	0.066514	0.032560	2.043

Random Effects

Groups	Name	Variance	Standard Deviation
Xr	S(Age)	0.0000	0.0000
Site	(Intercept)	0.1297	0.3601
Residual		0.4643	0.6814

Generalized Additive Model

Parametric coefficients

	Estimate	Std. Error	t-value	P-value
Intercept	-0.137220	0.192294	-0.714	0.4758
Scan Quality	0.086345	0.038522	2.241	0.0255
Sex (Male)	0.102049	0.075720	1.348	0.1784
Total Intracranial Volume	0.596887	0.037139	16.072	<0.0000000000002
Childhood Stress	-0.070158	0.032226	-2.177	0.0300
Positive Parenting Youth Report	0.005024	0.032618	0.154	0.8777
Childhood Stress * Pos. Parenting Youth Report	0.070110	0.030193	2.322	0.0207

Smoothing terms

Term	F	p-value
S(Age)	4.173	0.0416

R^2 (adjusted)

0.461

Model Output. This table depicts Generalized Additive Mixed Models (GAMM) model output, including both Linear Mixed-Effects Model (LMEM) and Generalized Additive Model (GAM) components. LMEM components include both fixed effects, i.e., standardized regression coefficients, standard errors, t-values, and p-values, as well as random effects of imaging site and a smoothing term for Age. GAM output includes estimates, standard errors, t-values, and p-values, as well as parameters for an Age smoothing term.

Caregiver-Report Parenting and Stress on Total Hippocampal Volume (GAMM)

The results of this GAMM are again consistent with the LMEM reported in the manuscript; namely, that using caregiver-reported positive parenting (instead of youth-reported positive parenting) results in a non-significant interaction between stress and positive parenting on total hippocampal volume. This supports our claim that centering youth-reports of parenting behaviors are important when considering the effects of stress and hippocampal development.

Caregiver-Report Parenting and Stress on Total Hippocampal Volume GAMM Linear Model Output

Model Title: Caregiver-Report Parenting and Stress on Total Hippocampal Volume (GAMM)

Observations: 480

Linear Mixed Effects Model

Fixed Effects

	Estimate	Std. Error	t-value
Intercept	-0.13368	0.19629	-0.681
Scan Quality	0.08376	0.03872	2.163
Sex (Male)	0.10574	0.07642	1.384
Total Intracranial Volume	0.59413	0.03720	15.970
Childhood Stress	-0.05759	0.03180	-1.811
Positive Parenting Caregiver Report	0.03649	0.03245	1.125
Childhood Stress * Pos. Parenting Youth Report	0.01555	0.03002	0.518
Age (smoothing term)	0.07586	0.03278	2.314

Random Effects

Groups	Name	Variance	Standard Deviation
Xr	S(Age)	0.0000	0.0000
Site	(Intercept)	0.1356	0.3683
Residual		0.4695	0.6852

Generalized Additive Model

Parametric coefficients

	Estimate	Std. Error	t-value	P-value
Intercept	-0.13368	0.19629	-0.681	0.4962
Scan Quality	0.13368	0.03872	2.163	0.0310
Sex (Male)	0.10574	0.07642	1.384	0.1671
Total Intracranial Volume	0.59413	0.03720	15.970	<0.0000000000000002
Childhood Stress	-0.05759	0.03245	-1.811	0.0707
Positive Parenting Caregiver Report	0.03649	0.03245	1.125	0.2613
Childhood Stress * Pos. Parenting Youth Report	0.01555	0.03002	0.518	0.6048

Smoothing terms

Term	F	p-value
S(Age)	5.355	0.0211

R^2 (adjusted)

0.454

Model Output. This table depicts Generalized Additive Mixed Models (GAMM) model output, including both Linear Mixed-Effects Model (LMEM) and Generalized Additive Model (GAM) components. LMEM components include both fixed effects, i.e., standardized regression coefficients, standard errors, t-values, and p-values, as well as random effects of imaging site and a smoothing term for Age. GAM output includes estimates, standard errors, t-values, and p-values, as well as parameters for an Age smoothing term.

Youth-Report Parenting and Stress on Total Amygdala Volume (GAMM)

The results of the next two GAMMs are consistent with the LMEs invesgiating total amygdala volume. Neither youth-reported positive parenting nor caregiver-reported positive parenting significantly interact with stress to predict total amygdala volume.

Model Title: Youth-Report Parenting and Stress on Total Amygdala Volume (GAMM)

Observations: 482

Linear Mixed Effects Model

Fixed Effects

	Estimate	Std. Error	t-value
Intercept	-0.31575	0.18564	-1.701
Scan Quality	0.10960	0.04039	2.714
Sex (Male)	0.30067	0.07951	3.781
Total Intracranial Volume	0.49545	0.03900	12.705
Childhood Stress	-0.03317	0.03384	-0.980
Positive Parenting Youth Report	-0.04019	0.03425	-1.173
Childhood Stress * Pos. Parenting Youth Report	0.01744	0.03170	0.550
Age (smoothing term)	0.07930	0.03419	2.319

Random Effects

Groups	Name	Variance	Standard Deviation
Xr	S(Age)	0.000	0.0000
Site	(Intercept)	0.118	0.3434
Residual		0.512	0.7156

Generalized Additive Model

Parametric coefficients

	Estimate	Std. Error	t-value	P-value
Intercept	-0.31575	0.18564	-1.701	0.089627
Scan Quality	0.10960	0.04039	2.714	0.006899
Sex (Male)	0.30067	0.07951	3.781	0.000176
Total Intracranial Volume	0.49545	0.03900	12.705	<0.0000000000000002
Childhood Stress	-0.03317	0.03384	-0.980	0.327489
Positive Parenting Youth Report	-0.04019	0.03425	-1.173	0.241237
Childhood Stress * Pos. Parenting Youth Report	0.01744	0.03170	0.550	0.582600

Smoothing terms

Term	F	p-value
S(Age)	5.379	0.0208

R^2 (adjusted)

0.417

Model Output. This table depicts Generalized Additive Mixed Models (GAMM) model output, including both Linear Mixed-Effects Model (LMEM) and Generalized Additive Model (GAM) components. LMEM components include both fixed effects, i.e., standardized regression coefficients, standard errors, t-values, and

p-values, as well as random effects of imaging site and a smoothing term for Age. GAM output includes estimates, standard errors, t-values, and p-values, as well as parameters for an Age smoothing term.

Caregiver-Report Parenting and Stress on Total Amygdala Volume (GAMM)

Model Title: Caregiver-Report Parenting and Stress on Total Amygdala Volume (GAMM)

Observations: 480

Linear Mixed Effects Model

Fixed Effects

	Estimate	Std. Error	t-value
Intercept	-0.314816	0.187410	-1.680
Scan Quality	0.113255	0.040490	2.797
Sex (Male)	0.302659	0.080057	3.781
Total Intracranial Volume	0.499088	0.038969	12.807
Childhood Stress	-0.031267	0.033311	-0.939
Positive Parenting Caregiver Report	-0.009294	0.033988	-0.273
Childhood Stress * Pos. Parenting Youth Report	-0.018973	0.031451	-0.603
Age (smoothing term)	0.086886	0.034342	2.530

Random Effects

Groups	Name	Variance	Standard Deviation
Xr	S(Age)	0.0000	0.0000
Site	(Intercept)	0.1204	0.3470
Residual		0.5153	0.7178

Generalized Additive Model

Parametric coefficients

	Estimate	Std. Error	t-value	P-value
Intercept	-0.314816	0.187410	-1.680	0.093653
Scan Quality	0.113255	0.040490	2.797	0.005367
Sex (Male)	0.302659	0.080057	3.781	0.000177
Total Intracranial Volume	0.499088	0.038969	12.807	< 2e-16
Childhood Stress	-0.031267	0.033311	-0.939	0.348386
Positive Parenting Caregiver Report	-0.009294	0.033988	-0.273	0.784619
Childhood Stress * Pos. Parenting Youth Report	-0.018973	0.031451	-0.603	0.546626

Smoothing terms

Term	F	p-value
S(Age)	6.401	0.0117

R^2 (adjusted)

0.414

Model Output. This table depicts Generalized Additive Mixed Models (GAMM) model output, including both Linear Mixed-Effects Model (LMEM) and Generalized Additive Model (GAM) components. LMEM components include both fixed effects, i.e., standardized regression coefficients, standard errors, t-values, and p-values, as well as random effects of imaging site and a smoothing term for Age. GAM output includes estimates, standard errors, t-values, and p-values, as well as parameters for an Age smoothing term.

Probing Questions of Interest Using a Quadtratic Stress Term to Account for Potential Non-Linear Stress Effects

We ran a series of GAMMs modeling stress as a quadratic variable and a spline/smooth function applied to the interaction between stress and age. For total amygdala volume, as well as right and left amygdala volume, we fit a GAMM with a random effect of site, a quadratic stress (i.e, negative life events) term, main effects of sex, estimated total intracranial volume ($Total.Intracranial.Vol$), youth-reported positive parenting, and a a smooth (non-linear) function applied to the interaction between negative life events and age. We also ran 3 additional models with the same DVs (ie.., total, left, and right amygdala) but instead using total Gray Matter Volume as the brain scaling covariate. Results indicate non-significant effects of stress and the interaction between stress and age. Notably, cross-sectional data is not well-suited to interrogate effects of interest (i.e., effects between stress and age) given that complex non-linear relations between amygdala structure and stress experiences play out over time.

Youth-Report Parenting and Non-Linear Stress on Total Amygdala Volume (GAMM)

Model Title: Youth-Report Parenting and Non-Linear Stress on Total Amygdala Volume (GAMM)

Observations: 482

Linear Mixed Effects Model

Fixed Effects

	Estimate	Std. Error	t-value
Intercept	- 0.32136	0.18768	- 1.723
Scan Quality	0.10653	0.04027	2.645
Sex (Male)	0.31033	0.07949	3.904
Total Intracranial Volume	0.49057	0.03917	12.525
Age	0.07773	0.03417	2.274
Childhood Stress (quadratic)	- 0.01316	0.03346	- 0.393
Positive Parenting Youth Report	- 0.04316	0.03416	-1.263
Non-linear Childhood Stress * Age (smoothing term)	-0.03114	0.03295	-0.945

Random Effects

Groups	Name	Variance	Standard Deviation
Xr	S(interaction)	0.0000	0.0000
Site	(Intercept)	0.1192	0.3452
Residual		0.5121	0.7156

Generalized Additive Model

Parametric coefficients

	Estimate	Std. Error	t-value	P-value
Intercept	- 0.32136	0.18647	- 1.723	0.085473
Scan Quality	0.10653	0.04027	2.645	0.008432
Sex (Male)	0.31033	0.07949	3.904	0.000108
Age	0.07773	0.03417	2.274	0.023382
Total Intracranial Volume	0.49057	0.03917	12.525	<0.0000000000000002
Childhood Stress (quadratic)	- 0.01316	0.03346	- 0.393	0.694260
Positive Parenting Youth Report	- 0.04316	0.03416	- 1.263	0.207039

Smoothing terms

Term	F	p-value
S(Age)	0.893	0.345

R^2 (adjusted)

0.416

Model Output. This table depicts Generalized Additive Mixed Models (GAMM) model output, including both Linear Mixed-Effects Model (LMEM) and Generalized Additive Model (GAM) components. LMEM components include both fixed effects, i.e., standardized regression coefficients, standard errors, t-values, and p-values, as well as random effects of imaging site and a smoothing term for Age. GAM output includes estimates, standard errors, t-values, and p-values, as well as parameters for an Age smoothing term.

Youth-Report Parenting and Non-Linear Stress on Left Amygdala Volume (GAMM)

Model Title: Youth-Report Parenting and Non-Linear Stress on Left Amygdala Volume (GAMM)

Observations: 482

Linear Mixed Effects Model

Fixed Effects

	Estimate	Std. Error	t-value
Intercept	-0.30855	0.16596	-1.859
Scan Quality	0.09804	0.04331	2.264
Sex (Male)	0.32846	0.08591	3.823
Age	0.09465	0.03693	2.563
Total Intracranial Volume	0.42663	0.04232	10.081
Childhood Stress (quadratic)	-0.03924	0.03617	-1.085
Positive Parenting Youth Report	-0.04622	0.03692	-1.252
Non-linear Childhood Stress * Age (smoothing term)	-0.03743	0.03561	-1.051

Random Effects

Groups	Name	Variance	Standard Deviation
Xr	S(interaction)	0.00000	0.0000
Site	(Intercept)	0.08761	0.2960
Residual		0.59832	0.7735

Generalized Additive Model

Parametric coefficients

	Estimate	Std. Error	t-value	P-value
Intercept	-0.30855	0.16596	-1.859	0.063621
Scan Quality	0.09804	0.04331	2.264	0.024052
Sex (Male)	0.32846	0.08591	3.823	0.000149
Age	0.09465	0.03693	2.563	0.010686
Total Intracranial Volume	0.42663	0.04232	10.081	<0.0000000000000002
Childhood Stress (quadratic)	-0.03924	0.03617	-1.085	0.278544
Positive Parenting Youth Report	-0.04622	0.03692	-1.252	0.211241

Smoothing terms

Term	F	p-value
S(Age)	1.104	0.294

R^2 (adjusted)

0.35

Model Output. This table depicts Generalized Additive Mixed Models (GAMM) model output, including both Linear Mixed-Effects Model (LMEM) and Generalized Additive Model (GAM) components. LMEM components include both fixed effects, i.e., standardized regression coefficients, standard errors, t-values, and p-values, as well as random effects of imaging site and a smoothing term for Age. GAM output includes estimates, standard errors, t-values, and p-values, as well as parameters for an Age smoothing term.

Youth-Report Parenting and Non-Linear Stress on Right Amygdala Volume (GAMM)

Model Title: Youth-Report Parenting and Non-Linear Stress on Right Amygdala Volume (GAMM)

Observations: 482

Linear Mixed Effects Model

Fixed Effects

	Estimate	Std. Error	t-value
Intercept	-0.292913	0.191805	-1.527
Scan Quality	0.107173	0.040929	2.619
Sex (Male)	0.257154	0.080757	3.184
Age (smoothing term)	0.054797	0.034798	1.575
Total Intracranial Volume	0.501771	0.039799	12.608
Childhood Stress (quadratic)	0.006143	0.034666	0.177
Positive Parenting Youth Report	-0.037111	0.034688	-1.070
Non-linear Childhood Stress * Age (smoothing term)	-0.018609	0.071843	-0.259

Random Effects

Groups	Name	Variance	Standard Deviation
Xr	S(interaction)	0.1936	0.4400
Site	(Intercept)	0.1266	0.3558
Residual		0.5274	0.7263

Generalized Additive Model

Parametric coefficients

	Estimate	Std. Error	t-value	P-value
Intercept	-0.292913	0.191805	-1.527	0.12739
Scan Quality	0.107173	0.040929	2.619	0.00911
Sex (Male)	0.257154	0.080757	3.184	0.00155
Age	0.054797	0.034798	1.575	0.11599
Total Intracranial Volume	0.501771	0.039799	12.608	<0.0000000000000002
Childhood Stress (quadratic)	0.006143	0.034666	0.177	0.85943
Positive Parenting Youth Report	-0.037111	0.034688	-1.070	0.28524

Smoothing terms

Term	F	p-value
S(Age)	0.965	0.486

R^2 (adjusted)

0.397

Model Output. This table depicts Generalized Additive Mixed Models (GAMM) model output, including both Linear Mixed-Effects Model (LMEM) and Generalized Additive Model (GAM) components. LMEM components include both fixed effects, i.e., standardized regression coefficients, standard errors, t-values, and p-values, as well as random effects of imaging site and a smoothing term for Age. GAM output includes estimates, standard errors, t-values, and p-values, as well as parameters for an Age smoothing term.

Youth-Report Parenting and Non-Linear Stress on Total Amygdala Volume using Total Gray Matter Volume as the Brain Covariate (GAMM)

Model Title: Youth-Report Parenting and Non-Linear Stress on Total Amygdala Volume using Total Gray Matter Volume as the Brain Covariate (GAMM)

Observations: 482

Linear Mixed Effects Model

Fixed Effects

	Estimate	Std. Error	t-value
Intercept	-0.26999	0.18097	-1.492
Scan Quality	0.05441	0.03887	0.03887
Sex (Male)	0.22358	0.07748	2.886
Age	0.25156	0.03359	7.489
Total Gray Matter Volume	0.56689	0.03919	14.466
Childhood Stress (quadratic)	-0.03029	0.03217	-0.941
Positive Parenting Youth Report	-0.03994	0.03281	-1.217
Non-linear Childhood Stress * Age (smoothing term)	-0.02871	0.03165	-0.907

Random Effects

Groups	Name	Variance	Standard Deviation
Xr	S(interaction)	0.0000	0.0000
Site	(Intercept)	0.1123	0.3351
Residual		0.4728	0.6876

Generalized Additive Model

Parametric coefficients

	Estimate	Std. Error	t-value	P-value
Intercept	-0.26999	0.18097	-1.492	0.13638
Scan Quality	0.05441	0.03887	1.400	0.16221
Sex (Male)	0.22358	0.07748	2.886	0.00408
Age	0.25156	0.03359	7.489	0.000000000000342
Total Gray Matter Volume	0.56689	0.03919	14.466	<0.0000000000000002
Childhood Stress (quadratic)	-0.03029	0.03217	-0.941	0.34698
Positive Parenting Youth Report	-0.03994	0.03281	-1.217	0.22414

Smoothing terms

Term	F	p-value
S(Age)	0.823	0.365

R^2 (adjusted)

0.46

Model Output. This table depicts Generalized Additive Mixed Models (GAMM) model output, including both Linear Mixed-Effects Model (LMEM) and Generalized Additive Model (GAM) components. LMEM components include both fixed effects, i.e., standardized regression coefficients, standard errors, t-values, and p-values, as well as random effects of imaging site and a smoothing term for Age. GAM output includes estimates, standard errors, t-values, and p-values, as well as parameters for an Age smoothing term.

Youth-Report Parenting and Non-Linear Stress on Left Amygdala Volume using Total Gray Matter Volume as the Brain Covariate (GAMM)

Model Title: Youth-Report Parenting and Non-Linear Stress on Left Amygdala Volume using Total Gray Matter Volume as the Brain Covariate (GAMM)

Observations: 482

Linear Mixed Effects Model

Fixed Effects

	Estimate	Std. Error	t-value
Intercept	-0.25197	0.15973	-1.577
Scan Quality	0.05129	0.04193	1.223
Sex (Male)	0.23237	0.08399	2.767
Age	0.24984	0.03641	6.861
Total Gray Matter Volume	0.51240	0.04247	12.066
Childhood Stress (quadratic)	-0.05472	0.03489	-1.569
Positive Parenting Youth Report	-0.04187	0.03558	-1.177
Non-linear Childhood Stress * Age (smoothing term)	-0.03384	0.03431	-0.986

Random Effects

Groups	Name	Variance	Standard Deviation
Xr	S(interaction)	0.0000	0.0000
Site	(Intercept)	0.0808	0.2843
Residual		0.5559	0.7456

Generalized Additive Model

Parametric coefficients

	Estimate	Std. Error	t-value	P-value
Intercept	-0.25197	0.15973	-1.577	0.11535
Scan Quality	0.05129	0.04193	1.223	0.22184
Sex (Male)	0.23237	0.08399	2.767	0.00588
Age	0.24984	0.03641	6.861	0.0000000000214
Total Gray Matter Volume	0.51240	0.04247	12.066	<0.0000000000000002
Childhood Stress (quadratic)	-0.05472	0.03489	-1.569	0.11743
Positive Parenting Youth Report	0.04187	0.03558	-1.177	0.23986

Smoothing terms

Term	F	p-value
S(Age)	0.972	0.325

R^2 (adjusted)

0.397

Model Output. This table depicts Generalized Additive Mixed Models (GAMM) model output, including both Linear Mixed-Effects Model (LMEM) and Generalized Additive Model (GAM) components. LMEM components include both fixed effects, i.e., standardized regression coefficients, standard errors, t-values, and p-values, as well as random effects of imaging site and a smoothing term for Age. GAM output includes estimates, standard errors, t-values, and p-values, as well as parameters for an Age smoothing term.

Youth-Report Parenting and Non-Linear Stress on Right Amygdala Volume using Total Gray Matter Volume as the Brain Covariate (GAMM)

Model Title: Youth-Report Parenting and Non-Linear Stress on Right Amygdala Volume using Total Gray Matter Volume as the Brain Covariate (GAMM)

Observations: 482

Linear Mixed Effects Model

Fixed Effects

	Estimate	Std. Error	t-value
Intercept	-0.25138	0.18724	-1.343
Scan Quality	0.05573	0.04004	1.392
Sex (Male)	0.18877	0.07978	2.366
Total Gray Matter Volume	0.56086	0.04035	13.899
Childhood Stress (quadratic)	-0.01056	0.03379	-0.313
Positive Parenting Youth Report	-0.03526	0.03378	-1.044
Non-linear Childhood Stress * Age	-0.01967	0.06935	-0.284
Age (smoothing term)	0.22862	0.03469	6.590

Random Effects

Groups	Name	Variance	Standard Deviation
Xr	S(interaction)	0.1791	0.4232
Site	(Intercept)	0.1204	0.3470
Residual		0.5004	0.7074

Generalized Additive Model

Parametric coefficients

	Estimate	Std. Error	t-value	P-value
Intercept	-0.25138	0.18724	-1.343	0.1801
Scan Quality	0.05573	0.04004	1.392	0.1646
Sex (Male)	0.18877	0.07978	2.366	0.0184
Age	0.22862	0.03469	6.590	0.000000000117
Total Gray Matter Volume	0.56086	0.04035	13.899	<0.0000000000000002
Childhood Stress (quadratic)	-0.01056	0.03379	-0.313	0.7547
Positive Parenting Youth Report	-0.03526	0.03378	-1.044	0.2971

Smoothing terms

Term	F	p-value
S(Age)	0.94	0.496

R^2 (adjusted)

0.427

Model Output. This table depicts Generalized Additive Mixed Models (GAMM) model output, including both Linear Mixed-Effects Model (LMEM) and Generalized Additive Model (GAM) components. LMEM components include both fixed effects, i.e., standardized regression coefficients, standard errors, t-values, and p-values, as well as random effects of imaging site and a smoothing term for Age. GAM output includes estimates, standard errors, t-values, and p-values, as well as parameters for an Age smoothing term.

Investigating Total Brain Region Volumes with Total Grey Matter Volume as an Alternative Covariate to Estimated Total Intracranial Volume

Our main models in the manuscript included estimated total intracranial volume (*eTIV* / *Total.Intracranial.Vol*) as our brain covariate to control for individual differences in brain size. We ran sensitivity analyses with an alternative measure of brain size, *Total Gray Matter Volume* (Total.Gray.Vol) for each of our main LMEMs. Overall, we found that the inclusion of *Total.Gray.Vol* versus *Total.Intracranial.Vol* does not significantly impact model results.

In parallel to the main models presented in the manuscript, there remained a significant interaction between youth-reported parenting and stress on changes in total hippocampal volume when *Total.Gray.Vol* was included as the brain scaling variable instead of *Total.Intracranial.Vol*. These sensitivity analyses underscore that the significant interaction between stress and youth-reported positive parenting on hippocampal volumes is consistent even when accounting for individual differences in brain size in various ways. As in our main models, there was not a significant association between the interaction of caregiver-reported parenting and stress on hippocampal volume when *Total.Gray.Vol* was included as the brain scaling variable instead of *Total.Intracranial.Vol*. These findings continue to highlight importance of centering *youth* reports to understand associations between stress, parenting, and the hippocampus.

Aligned with the main models, the inclusion of *Total.Gray.Vol* instead of *Total.Intracranial.Vol* in models with the amygdala did not change results (i.e., associations remained non-significant).

Total.Gray.Vol, Youth-Report Parenting, and Stress on Total Hippocampal Volume (LMEM)

Observations	482
Dependent variable	TotalHippocampalVolume
Type	Mixed effects linear regression

AIC	1016.7240
BIC	1058.5035
Pseudo-R^2 (fixed effects)	0.4602
Pseudo-R^2 (total)	0.5818

Fixed Effects					
	Est.	S.E.	t val.	d.f.	p
(Intercept)	-0.0876	0.1876	-0.4668	3.3969	0.6689
Scan.Quality	0.0254	0.0370	0.6847	467.8754	0.4939
SexMale	0.0208	0.0735	0.2829	471.6270	0.7774
Age	0.2737	0.0319	8.5749	471.6079	0.0000
Total.Gray.Vol	0.6676	0.0371	18.0165	472.5797	0.0000
Childhood.Stress	-0.0898	0.0309	-2.9058	471.1317	0.0038
Pos.Parenting.Youth.Report	0.0064	0.0312	0.2044	471.1925	0.8381
Childhood.Stress:Pos.Parenting.Youth.Report	0.0692	0.0289	2.3948	471.9810	0.0170

p values calculated using Satterthwaite d.f.

Random Effects		
Group	Parameter	Std. Dev.
Site	(Intercept)	0.3518
Residual		0.6525

Grouping Variables		
Group	# groups	ICC
Site	4	0.2253

Model Output. This table depicts mixed effects linear regression model output, including both fixed effects and random effects. Fixed effects include standardized regression coefficients (Est.), standard errors (S.E.), t-values (t val.), and p-values (p-val). Random effects include the random effect of Imaging Site and the number of grouping variables (4 imaging sites). Model output also depicts overall model fit statistics and number of observations.

Total.Gray.Vol, Caregiver-Report Parenting, and Stress on Total Hippocampal Volume (LMEM)

Observations	480
Dependent variable	TotalHippocampalVolume
Type	Mixed effects linear regression

AIC	1017.9482
BIC	1059.6861
Pseudo-R^2 (fixed effects)	0.4546
Pseudo-R^2 (total)	0.5802

Fixed Effects					
	Est.	S.E.	t val.	d.f.	p
(Intercept)	-0.0824	0.1910	-0.4314	3.3871	0.6922
Scan.Quality	0.0243	0.0372	0.6514	466.2246	0.5151
SexMale	0.0200	0.0743	0.2690	469.5849	0.7881
Age	0.2836	0.0319	8.8798	469.5467	0.0000
Total.Gray.Vol	0.6663	0.0372	17.9181	470.6062	0.0000
Childhood.Stress	-0.0776	0.0305	-2.5461	469.1329	0.0112
Pos.Parenting.Caregiver.Report	0.0445	0.0311	1.4324	469.7739	0.1527
Childhood.Stress:Pos.Parenting.Caregiver.Report	0.0156	0.0287	0.5441	469.1305	0.5866

p values calculated using Satterthwaite d.f.

Random Effects		
Group	Parameter	Std. Dev.
Site	(Intercept)	0.3588
Residual		0.6560

Grouping Variables		
Group	# groups	ICC
Site	4	0.2303

Model Output. This table depicts mixed effects linear regression model output, including both fixed effects and random effects. Fixed effects include standardized regression coefficients (Est.), standard errors (S.E.), t-values (t val.), and p-values (p-val). Random effects include the random effect of Imaging Site and the number of grouping variables (4 imaging sites). Model output also depicts overall model fit statistics and number of observations.

Total.Gray.Vol, Youth-Report Parenting, and Stress on Total Amygdala Volume (LMEM)

Observations	482
Dependent variable	TotalAmygdalaVolume
Type	Mixed effects linear regression

AIC	1065.0284
BIC	1106.8079
Pseudo-R^2 (fixed effects)	0.4220
Pseudo-R^2 (total)	0.5319

Fixed Effects					
	Est.	S.E.	t val.	d.f.	p
(Intercept)	-0.2641	0.1799	-1.4682	3.5456	0.2247
Scan.Quality	0.0575	0.0389	1.4771	462.6587	0.1403
SexMale	0.2137	0.0774	2.7605	471.8043	0.0060
Age	0.2551	0.0336	7.5909	471.7844	0.0000
Total.Gray.Vol	0.5724	0.0390	14.6738	472.8545	0.0000
Childhood.Stress	-0.0504	0.0325	-1.5479	471.2167	0.1223
Pos.Parenting.Youth.Report	-0.0376	0.0329	-1.1427	471.2897	0.2537
Childhood.Stress:Pos.Parenting.Youth.Report	0.0171	0.0304	0.5623	472.1763	0.5742

p values calculated using Satterthwaite d.f.

Random Effects		
Group	Parameter	Std. Dev.
Site	(Intercept)	0.3329
Residual		0.6870

Grouping Variables		
Group	# groups	ICC
Site	4	0.1902

Model Output. This table depicts mixed effects linear regression model output, including both fixed effects and random effects. Fixed effects include standardized regression coefficients (Est.), standard errors (S.E.), t-values (t val.), and p-values (p-val). Random effects include the random effect of Imaging Site and the number of grouping variables (4 imaging sites). Model output also depicts overall model fit statistics and number of observations.

Total.Gray.Vol, Caregiver-Report Parenting, and Stress on Total Amygdala Volume (LMEM)

Observations	480
Dependent variable	TotalAmygdalaVolume
Type	Mixed effects linear regression

AIC	1063.9262
BIC	1105.6641
Pseudo-R^2 (fixed effects)	0.4200
Pseudo-R^2 (total)	0.5311

Fixed Effects					
	Est.	S.E.	t val.	d.f.	p
(Intercept)	-0.2620	0.1812	-1.4459	3.5503	0.2303
Scan.Quality	0.0618	0.0391	1.5834	460.6539	0.1140
SexMale	0.2125	0.0781	2.7221	469.7741	0.0067
Age	0.2648	0.0336	7.8898	469.7311	0.0000
Total.Gray.Vol	0.5766	0.0391	14.7605	470.9100	0.0000
Childhood.Stress	-0.0487	0.0320	-1.5220	469.2264	0.1287
Pos.Parenting.Caregiver.Report	-0.0021	0.0326	-0.0650	469.9559	0.9482
Childhood.Stress:Pos.Parenting.Caregiver.Report	-0.0188	0.0302	-0.6241	469.2238	0.5329

p values calculated using Satterthwaite d.f.

Random Effects		
Group	Parameter	Std. Dev.
Site	(Intercept)	0.3356
Residual		0.6892

Grouping Variables		
Group	# groups	ICC
Site	4	0.1916

Model Output. This table depicts mixed effects linear regression model output, including both fixed effects and random effects. Fixed effects include standardized regression coefficients (Est.), standard errors (S.E.), t-values (t val.), and p-values (p-val). Random effects include the random effect of Imaging Site and the number of grouping variables (4 imaging sites). Model output also depicts overall model fit statistics and number of observations.

Investigating Left and Right Hemispheres of Brain Regions Separately

Our main models in the manuscript investigated the interactions between childhoos stress and both youth- and caregiver-reported positive parenting on *total* hippocampal and amygdala volume. The *total* volume variables were derived by adding the volumes for the left and right hemispheres of the hippocampus and amygdala, respectively. We ran sensitivity analyses investigating the interactions between childhood stress and both youth- and caregiver-reported positive parenting on each the left and right hemisphere of the hippocampus and amygdala. Briefly, we found that the interaction between stress and youth-reported positive parenting was significant for the left *and* right hippocampus, and was not significant for either the left or right amygdala. Consistent with the pattern of results reported in our manuscript, the interaction between childhood stress and caregiver-reported positive parenting was not significant for the left hippocampus, right hippocampus, left amygdala, or right amydala.

These analyses underscore that, when centering the youth's perspective of positive parenting, there is a consistent buffering effect of positive parenting on the association between stress and hippocampal volumes, whether that be total volume, left hemisphere, or right hemisphere. Our models do not provide evidence for an analogous effect for the amgydala.

Youth-Report Parenting and Stress on L and R Hippocampal Hemispheres Separately (LMEM)

Left Hippocampus

Observations	482
Dependent variable	Left.Hippocampus
Type	Mixed effects linear regression

AIC	1083.8338
BIC	1125.6133
Pseudo-R^2 (fixed effects)	0.4287
Pseudo-R^2 (total)	0.5094

Fixed Effects					
	Est.	S.E.	t val.	d.f.	p
(Intercept)	-0.1367	0.1578	-0.8662	3.6858	0.4391
Scan.Quality	0.0998	0.0395	2.5291	445.9262	0.0118
SexMale	0.1047	0.0779	1.3438	471.7533	0.1797
Age	0.0656	0.0335	1.9561	471.8870	0.0510
Total.Intracranial.Vol	0.5996	0.0382	15.6920	472.6641	0.0000
Childhood.Stress	-0.0789	0.0332	-2.3775	471.2844	0.0178
Pos.Parenting.Youth.Report	0.0086	0.0336	0.2573	471.4025	0.7971
Childhood.Stress:Pos.Parenting.Youth.Report	0.0710	0.0311	2.2849	472.4689	0.0228

p values calculated using Satterthwaite d.f.

Random Effects		
Group	Parameter	Std. Dev.
Site	(Intercept)	0.2844
Residual		0.7014

Grouping Variables		
Group	# groups	ICC
Site	4	0.1412

Model Output. This table depicts mixed effects linear regression model output, including both fixed effects and random effects. Fixed effects include standardized regression coefficients (Est.), standard errors (S.E.), t-values (t val.), and p-values (p-val). Random effects include the random effect of Imaging Site and the number of grouping variables (4 imaging sites). Model output also depicts overall model fit statistics and number of observations.

Right Hippocampus

Observations	482
Dependent variable	Right.Hippocampus
Type	Mixed effects linear regression

AIC	1090.2878
BIC	1132.0672
Pseudo-R^2 (fixed effects)	0.3560
Pseudo-R^2 (total)	0.5210

Fixed Effects					
	Est.	S.E.	t val.	d.f.	p
(Intercept)	-0.1289	0.2184	-0.5904	3.3065	0.5929
Scan.Quality	0.0704	0.0399	1.7653	470.4692	0.0782
SexMale	0.0940	0.0783	1.2006	471.3477	0.2305
Age	0.0636	0.0337	1.8857	471.4505	0.0599
Total.Intracranial.Vol	0.5600	0.0384	14.5787	471.9897	0.0000
Childhood.Stress	-0.0580	0.0333	-1.7398	471.0945	0.0826
Pos.Parenting.Youth.Report	0.0012	0.0337	0.0356	471.1545	0.9716
Childhood.Stress:Pos.Parenting.Youth.Report	0.0654	0.0312	2.0947	471.8558	0.0367

p values calculated using Satterthwaite d.f.

Random Effects		
Group	Parameter	Std. Dev.
Site	(Intercept)	0.4136
Residual		0.7047

Grouping Variables		
Group	# groups	ICC
Site	4	0.2562

Model Output. This table depicts mixed effects linear regression model output, including both fixed effects and random effects. Fixed effects include standardized regression coefficients (Est.), standard errors (S.E.), t-values (t val.), and p-values (p-val). Random effects include the random effect of Imaging Site and the number of grouping variables (4 imaging sites). Model output also depicts overall model fit statistics and number of observations.

Youth-Report Parenting and Stress on L and R Amygdala Hemispheres Separately (LMEM)

Left Amygdala

Observations	482
Dependent variable	Left.Amygdala
Type	Mixed effects linear regression

AIC	1177.2170
BIC	1218.9965
Pseudo-R^2 (fixed effects)	0.3200
Pseudo-R^2 (total)	0.4060

Fixed Effects					
	Est.	S.E.	t val.	d.f.	p
(Intercept)	-0.3057	0.1653	-1.8493	3.7979	0.1419
Scan.Quality	0.0998	0.0435	2.2946	437.2392	0.0222
SexMale	0.3258	0.0860	3.7869	471.8579	0.0002
Age	0.0951	0.0370	2.5696	471.9919	0.0105
Total.Intracranial.Vol	0.4316	0.0422	10.2332	472.7956	0.0000
Childhood.Stress	-0.0391	0.0366	-1.0670	471.3441	0.2865
Pos.Parenting.Youth.Report	-0.0475	0.0371	-1.2811	471.4754	0.2008
Childhood.Stress:Pos.Parenting.Youth.Report	0.0022	0.0343	0.0643	472.5926	0.9488

p values calculated using Satterthwaite d.f.

Random Effects		
Group	Parameter	Std. Dev.
Site	(Intercept)	0.2945
Residual		0.7743

Grouping Variables		
Group	# groups	ICC
Site	4	0.1264

Model Output. This table depicts mixed effects linear regression model output, including both fixed effects and random effects. Fixed effects include standardized regression coefficients (Est.), standard errors (S.E.), t-values (t val.), and p-values (p-val). Random effects include the random effect of Imaging Site and the number of grouping variables (4 imaging sites). Model output also depicts overall model fit statistics and number of observations.

Right Amygdala

Observations	482
Dependent variable	Right.Amygdala
Type	Mixed effects linear regression

AIC	1118.6052
BIC	1160.3847
Pseudo-R^2 (fixed effects)	0.3563
Pseudo-R^2 (total)	0.4790

Fixed Effects					
	Est.	S.E.	t val.	d.f.	p
(Intercept)	-0.2883	0.1903	-1.5147	3.5469	0.2132
Scan.Quality	0.1100	0.0410	2.6817	462.6412	0.0076
SexMale	0.2460	0.0808	3.0460	471.5807	0.0024
Age	0.0561	0.0348	1.6131	471.7008	0.1074
Total.Intracranial.Vol	0.5039	0.0396	12.7209	472.3555	0.0000
Childhood.Stress	-0.0242	0.0344	-0.7038	471.2368	0.4819
Pos.Parenting.Youth.Report	-0.0293	0.0348	-0.8427	471.3206	0.3998
Childhood.Stress:Pos.Parenting.Youth.Report	0.0302	0.0322	0.9368	472.1925	0.3493

p values calculated using Satterthwaite d.f.

Random Effects		
Group	Parameter	Std. Dev.
Site	(Intercept)	0.3527
Residual		0.7269

Grouping Variables		
Group	# groups	ICC
Site	4	0.1905

Model Output. This table depicts mixed effects linear regression model output, including both fixed effects and random effects. Fixed effects include standardized regression coefficients (Est.), standard errors (S.E.), t-values (t val.), and p-values (p-val). Random effects include the random effect of Imaging Site and the number of grouping variables (4 imaging sites). Model output also depicts overall model fit statistics and number of observations.

Caregiver-Report Parenting and Stress on L and R Hippocampal Hemispheres Separately (LMEM)

Left Hippocampus

Observations	480
Dependent variable	Left.Hippocampus
Type	Mixed effects linear regression

AIC	1084.1325
BIC	1125.8703
Pseudo-R^2 (fixed effects)	0.4223
Pseudo-R^2 (total)	0.5071

Fixed Effects					
	Est.	S.E.	t val.	d.f.	p
(Intercept)	-0.1351	0.1616	-0.8359	3.6582	0.4543
Scan.Quality	0.0965	0.0396	2.4361	445.9245	0.0152
SexMale	0.1117	0.0786	1.4207	469.6841	0.1561
Age	0.0748	0.0337	2.2197	469.5993	0.0269
Total.Intracranial.Vol	0.5956	0.0382	15.5726	470.6647	0.0000
Childhood.Stress	-0.0651	0.0327	-1.9894	469.2785	0.0472
Pos.Parenting.Caregiver.Report	0.0389	0.0334	1.1673	470.1312	0.2437
Childhood.Stress:Pos.Parenting.Caregiver.Report	0.0060	0.0309	0.1931	469.2593	0.8470

p values calculated using Satterthwaite d.f.

Random Effects		
Group	Parameter	Std. Dev.
Site	(Intercept)	0.2923
Residual		0.7047

Grouping Variables		
Group	# groups	ICC
Site	4	0.1468

Model Output. This table depicts mixed effects linear regression model output, including both fixed effects and random effects. Fixed effects include standardized regression coefficients (Est.), standard errors (S.E.), t-values (t val.), and p-values (p-val). Random effects include the random effect of Imaging Site and the number of grouping variables (4 imaging sites). Model output also depicts overall model fit statistics and number of observations.

Right Hippocampus

Observations	480
Dependent variable	Right.Hippocampus
Type	Mixed effects linear regression

AIC	1090.8136
BIC	1132.5515
Pseudo-R^2 (fixed effects)	0.3505
Pseudo-R^2 (total)	0.5202

Fixed Effects					
	Est.	S.E.	t val.	d.f.	p
(Intercept)	-0.1239	0.2221	-0.5579	3.2970	0.6126
Scan.Quality	0.0686	0.0401	1.7127	468.6376	0.0874
SexMale	0.0944	0.0790	1.1953	469.3129	0.2326
Age	0.0724	0.0339	2.1370	469.2737	0.0331
Total.Intracranial.Vol	0.5586	0.0385	14.5257	469.9959	0.0000
Childhood.Stress	-0.0473	0.0329	-1.4387	469.0982	0.1509
Pos.Parenting.Caregiver.Report	0.0322	0.0335	0.9591	469.6538	0.3380
Childhood.Stress:Pos.Parenting.Caregiver.Report	0.0237	0.0310	0.7631	469.0861	0.4458

p values calculated using Satterthwaite d.f.

Random Effects		
Group	Parameter	Std. Dev.
Site	(Intercept)	0.4212
Residual		0.7082

Grouping Variables		
Group	# groups	ICC
Site	4	0.2613

Model Output. This table depicts mixed effects linear regression model output, including both fixed effects and random effects. Fixed effects include standardized regression coefficients (Est.), standard errors (S.E.), t-values (t val.), and p-values (p-val). Random effects include the random effect of Imaging Site and the number of grouping variables (4 imaging sites). Model output also depicts overall model fit statistics and number of observations.

Caregiver-Report Parenting and Stress on L and R Amygdala Hemispheres Separately (LMEM)

Left Amygdala

Observations	480
Dependent variable	Left.Amygdala
Type	Mixed effects linear regression

AIC	1174.0209
BIC	1215.7588
Pseudo-R^2 (fixed effects)	0.3200
Pseudo-R^2 (total)	0.4060

Fixed Effects					
	Est.	S.E.	t val.	d.f.	p
(Intercept)	-0.3028	0.1656	-1.8282	3.8062	0.1452
Scan.Quality	0.1078	0.0435	2.4764	434.3002	0.0137
SexMale	0.3212	0.0865	3.7144	469.8195	0.0002
Age	0.1032	0.0371	2.7830	469.7193	0.0056
Total.Intracranial.Vol	0.4386	0.0421	10.4235	470.8460	0.0000
Childhood.Stress	-0.0405	0.0360	-1.1248	469.3549	0.2613
Pos.Parenting.Caregiver.Report	-0.0135	0.0367	-0.3683	470.2697	0.7128
Childhood.Stress:Pos.Parenting.Caregiver.Report	-0.0166	0.0340	-0.4888	469.3342	0.6252

p values calculated using Satterthwaite d.f.

Random Effects		
Group	Parameter	Std. Dev.
Site	(Intercept)	0.2950
Residual		0.7755

Grouping Variables		
Group	# groups	ICC
Site	4	0.1264

Model Output. This table depicts mixed effects linear regression model output, including both fixed effects and random effects. Fixed effects include standardized regression coefficients (Est.), standard errors (S.E.), t-values (t val.), and p-values (p-val). Random effects include the random effect of Imaging Site and the number of grouping variables (4 imaging sites). Model output also depicts overall model fit statistics and number of observations.

Right Amygdala

Observations	480
Dependent variable	Right.Amygdala
Type	Mixed effects linear regression

AIC	1116.3797
BIC	1158.1176
Pseudo-R^2 (fixed effects)	0.3522
Pseudo-R^2 (total)	0.4787

Fixed Effects					
	Est.	S.E.	t val.	d.f.	p
(Intercept)	-0.2895	0.1933	-1.4978	3.5433	0.2174
Scan.Quality	0.1093	0.0411	2.6583	461.1940	0.0081
SexMale	0.2539	0.0812	3.1255	469.5418	0.0019
Age	0.0623	0.0348	1.7875	469.4841	0.0745
Total.Intracranial.Vol	0.5040	0.0395	12.7443	470.3688	0.0000
Childhood.Stress	-0.0194	0.0338	-0.5724	469.2472	0.5673
Pos.Parenting.Caregiver.Report	-0.0043	0.0345	-0.1254	469.9397	0.9002
Childhood.Stress:Pos.Parenting.Caregiver.Report	-0.0192	0.0319	-0.6012	469.2318	0.5480

p values calculated using Satterthwaite d.f.

Random Effects		
Group	Parameter	Std. Dev.
Site	(Intercept)	0.3588
Residual		0.7285

Grouping Variables		
Group	# groups	ICC
Site	4	0.1952

Model Output. This table depicts mixed effects linear regression model output, including both fixed effects and random effects. Fixed effects include standardized regression coefficients (Est.), standard errors (S.E.), t-values (t val.), and p-values (p-val). Random effects include the random effect of Imaging Site and the number of grouping variables (4 imaging sites). Model output also depicts overall model fit statistics and number of observations.

Examining Associations Between Hippocampal Volumes and Youth Behavioral Problems

Our previous results found significant interactions between stress and positive parenting (from the youth perspective) on left, right, and total hippocampal volume. We lastly investigated the association between total hippocampal volume and youth behavioral problems. We considered both YSR Total Score (Youth-reported behavioral problems) and SDQ (caregiver-reported behavioral problems) as dependent variables in separate models.

We ran analyses considering YSR Total Score as linear mixed-effects models (LMEM) and as linear models (LM) due to issues with singularity for YSR Total Score in a LMEM. Across both types of models, results were consistent, with a non-significant relationship between total hippocampal volume and the YSR. We also examined associations with left and right hippocampal volumes, finding a significant association youth behavioral problems (YSR) and left hippocampal volumes.

Total Hippocampal Volumes on Youth Behavioral Problems (Youth-Report/YSR) as a linear mixed-effects model

```
## boundary (singular) fit: see help('isSingular')
```

Observations	410
Dependent variable	Youth.Self.Report.Total
Type	Mixed effects linear regression

AIC	1184.8705
BIC	1216.9997
Pseudo-R^2 (fixed effects)	0.0382
Pseudo-R^2 (total)	0.0382

Fixed Effects					
	Est.	S.E.	t val.	d.f.	p
(Intercept)	0.0792	0.0884	0.8953	404.0000	0.3712
SexMale	-0.2205	0.1202	-1.8349	404.0000	0.0673
Age	0.1783	0.0593	3.0050	404.0000	0.0028
Total.Intracranial.Vol	0.1042	0.0683	1.5251	404.0000	0.1280
Scan.Quality	0.0576	0.0515	1.1187	404.0000	0.2639
TotalHippocampalVolume	-0.0147	0.0666	-0.2209	404.0000	0.8253

p values calculated using Satterthwaite d.f.

Random Effects		
Group	Parameter	Std. Dev.
Site	(Intercept)	0.0000
Residual		0.9865

Grouping Variables		
Group	# groups	ICC
Site	4	0.0000

Model Output. This table depicts mixed effects linear regression model output, including both fixed effects and random effects. Fixed effects include standardized regression coefficients (Est.), standard errors (S.E.), t-values (t val.), and p-values (p-val). Random effects include the random effect of Imaging Site and the number of grouping variables (4 imaging sites). Model output also depicts overall model fit statistics and number of observations.

Total Hippocampal Volumes on Youth Behavioral Problems (Youth-Report/YSR) as a linear model

Observations	410 (72 missing obs. deleted)
Dependent variable	Youth.Self.Report.Total
Type	OLS linear regression

F(8,401)	2.1630
R^2	0.0414
Adj. R^2	0.0222

	Est.	S.E.	t val.	p
(Intercept)	0.0716	0.1020	0.7019	0.4831
SexMale	-0.2197	0.1209	-1.8163	0.0701
Age	0.1648	0.0610	2.7022	0.0072
Total.Intracranial.Vol	0.0948	0.0700	1.3528	0.1769
SiteCUNY	-0.1547	0.2969	-0.5209	0.6027
SiteRU	-0.0186	0.1165	-0.1599	0.8730
SiteSI	0.1485	0.1711	0.8678	0.3860
Scan.Quality	0.0793	0.0594	1.3337	0.1831
TotalHippocampalVolume	0.0069	0.0706	0.0973	0.9226

Standard errors: OLS

Model Output. This table depicts mixed effects linear regression model output, including both fixed effects and random effects. Fixed effects include standardized regression coefficients (Est.), standard errors (S.E.), t-values (t val.), and p-values (p-val). Random effects include the random effect of Imaging Site and the number of grouping variables (4 imaging sites). Model output also depicts overall model fit statistics and number of observations.

Total Hippocampal Volumes on Youth Behavioral Problems (Caregiver-Report/SDQ) as a linear mixed-effects model

We did not find a significant relationship between SDQ total score and total hippocampal volume.

Observations	404
Dependent variable	Strengths.Difficulties.Total
Type	Mixed effects linear regression

AIC	1172.3909
BIC	1204.4022
Pseudo-R^2 (fixed effects)	0.0267
Pseudo-R^2 (total)	0.0330

Fixed Effects					
	Est.	S.E.	t val.	d.f.	p
(Intercept)	-0.0513	0.1015	-0.5051	6.7294	0.6296
SexMale	0.1639	0.1220	1.3435	397.9778	0.1799
Age	-0.1148	0.0602	-1.9087	376.0531	0.0571
Total.Intracranial.Vol	0.0886	0.0691	1.2818	380.2227	0.2007
Scan.Quality	0.0870	0.0545	1.5968	74.5588	0.1145
TotalHippocampalVolume	-0.1055	0.0682	-1.5473	285.0808	0.1229

p values calculated using Satterthwaite d.f.

Random Effects		
Group	Parameter	Std. Dev.
Site	(Intercept)	0.0802
Residual		0.9908

Grouping Variables		
Group	# groups	ICC
Site	4	0.0065

Model Output. This table depicts mixed effects linear regression model output, including both fixed effects and random effects. Fixed effects include standardized regression coefficients (Est.), standard errors (S.E.), t-values (t val.), and p-values (p-val). Random effects include the random effect of Imaging Site and the number of grouping variables (4 imaging sites). Model output also depicts overall model fit statistics and number of observations.

Left Hippocampus on Youth Behavioral Problems (Caregiver-Report/SDQ) as a linear mixed-effects model

The association between the left hippocampus and total SDQ score was significant, indicating that increased youth behavioral problems are significantly associated with decreased volume in the left hippocampus. These results are visualized after the model output below. We did not find parallel results for the right hippocampus.

Observations	404
Dependent variable	Strengths.Difficulties.Total
Type	Mixed effects linear regression

AIC	1171.0211
BIC	1203.0325
Pseudo-R^2 (fixed effects)	0.0301
Pseudo-R^2 (total)	0.0354

Fixed Effects					
	Est.	S.E.	t val.	d.f.	p
(Intercept)	-0.0584	0.0997	-0.5859	7.0333	0.5763
SexMale	0.1718	0.1219	1.4095	397.9857	0.1595
Age	-0.1121	0.0600	-1.8674	373.8271	0.0626
Total.Intracranial.Vol	0.1038	0.0689	1.5070	391.1419	0.1326
Scan.Quality	0.0907	0.0540	1.6815	61.1981	0.0978
Left.Hippocampus	-0.1325	0.0675	-1.9625	339.4105	0.0505

p values calculated using Satterthwaite d.f.

Random Effects		
Group	Parameter	Std. Dev.
Site	(Intercept)	0.0734
Residual		0.9892

Grouping Variables		
Group	# groups	ICC
Site	4	0.0055

Model Output. This table depicts mixed effects linear regression model output, including both fixed effects and random effects. Fixed effects include standardized regression coefficients (Est.), standard errors (S.E.), t-values (t val.), and p-values (p-val). Random effects include the random effect of Imaging Site and the number of grouping variables (4 imaging sites). Model output also depicts overall model fit statistics and number of observations.

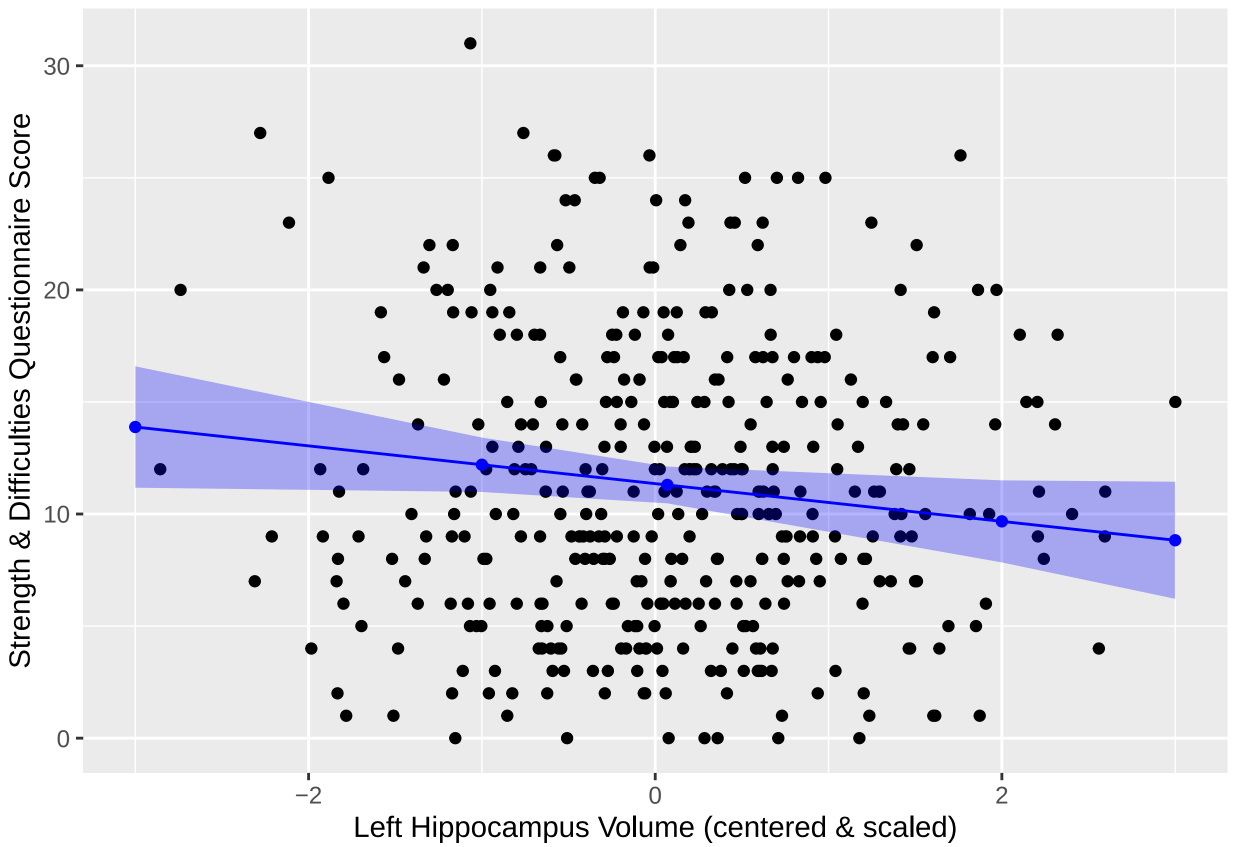

Figure S1. Association between left hippocampal volumes and Total score on the caregiver-reported Strengths & Difficulties Questionnaire.

Right Hippocampus on Youth Behavioral Problems (Caregiver-Report/SDQ) as a linear mixed-effects model

Observations	404
Dependent variable	Strengths.Difficulties.Total
Type	Mixed effects linear regression

AIC	1173.8087
BIC	1205.8200
Pseudo-R^2 (fixed effects)	0.0238
Pseudo-R^2 (total)	0.0327

Fixed Effects					
	Est.	S.E.	t val.	d.f.	p
(Intercept)	-0.0391	0.1057	-0.3697	5.9130	0.7245
SexMale	0.1527	0.1219	1.2520	397.7575	0.2113
Age	-0.1180	0.0603	-1.9555	379.6189	0.0513
Total.Intracranial.Vol	0.0635	0.0672	0.9445	380.1467	0.3455
Scan.Quality	0.0801	0.0549	1.4587	87.9534	0.1482
Right.Hippocampus	-0.0641	0.0654	-0.9801	264.9080	0.3279

p values calculated using Satterthwaite d.f.

Random Effects		
Group	Parameter	Std. Dev.
Site	(Intercept)	0.0953
Residual		0.9920

Grouping Variables		
Group	# groups	ICC
Site	4	0.0091

Model Output. This table depicts mixed effects linear regression model output, including both fixed effects and random effects. Fixed effects include standardized regression coefficients (Est.), standard errors (S.E.), t-values (t val.), and p-values (p-val). Random effects include the random effect of Imaging Site and the number of grouping variables (4 imaging sites). Model output also depicts overall model fit statistics and number of observations.

Comparing Key Variables between HBN-Imaging Sample to HBN-Total Sample

This data is drawn from the Healthy Brain Network (HBN) study, a large-scale, publicly-available dataset with measures from over 4,000 children across New York City. Importantly, not all participants have completed all measures. The total amount of datapoints for relevant study variables ranges from 482 to 3,437 depending on a) whether participants were part of the imaging protocol and b) missingness on study variables.

We ran missing data analyses comparing the subsample with imaging data (N = 482) to the larger HBN sample on the following key study variables: Youth-Reported Negative Life Events, Youth-Reported Positive Parenting, Caregiver-Reported Positive Parenting, and Youth Self-Report scores. We created a binary indicator variable *imaging* that we used as the independent variable in all below analyses to test for any differences between the imaging subsample and larger HBN sample (0 = not part of imaging sample, 1 = part of imaging sample).

For more information on the Healthy Brain Network or to get access to data, visit this site: http://fcon_1000.projects.nitrc.org/indi/cmi_healthy_brain_network/

Negative Life Events

Observations	999 (3161 missing obs. deleted)
Dependent variable	Childhood.Stress
Type	OLS linear regression

F(1,997)	1.4697
R^2	0.0015
Adj. R^2	0.0005

	Est.	S.E.	t val.	p
(Intercept)	2.9285	0.0504	58.0848	0.0000
imaging	-0.0880	0.0726	-1.2123	0.2257

Standard errors: OLS

Model Output. This table depicts linear regression model output, including standardized regression coefficients (Est.), standard errors (S.E.), t-values (t val.), and p-values (p-val). Model output also depicts overall model fit statistics and number of observations.

Youth-Report Parenting

Observations	3387 (773 missing obs. deleted)
Dependent variable	Pos.Parenting.Youth.Report
Type	OLS linear regression

F(1,3385)	19.9666
R^2	0.0059
Adj. R^2	0.0056

	Est.	S.E.	t val.	p
(Intercept)	22.4764	0.0912	246.4776	0.0000
imaging	-1.0802	0.2417	-4.4684	0.0000

Standard errors: OLS

Model Output. This table depicts linear regression model output, including standardized regression coefficients (Est.), standard errors (S.E.), t-values (t val.), and p-values (p-val). Model output also depicts overall model fit statistics and number of observations.

Caregiver-Report Parenting

Observations	3437 (723 missing obs. deleted)
Dependent variable	Pos.Parenting.Caregiver.Report
Type	OLS linear regression

F(1,3435)	30.1143
R^2	0.0087
Adj. R^2	0.0084

	Est.	S.E.	t val.	p
(Intercept)	25.8167	0.0572	451.5152	0.0000
imaging	-0.8396	0.1530	-5.4877	0.0000

Standard errors: OLS

Model Output. This table depicts linear regression model output, including standardized regression coefficients (Est.), standard errors (S.E.), t-values (t val.), and p-values (p-val). Model output also depicts overall model fit statistics and number of observations.

Youth-Self Report

Observations	1417 (2743 missing obs. deleted)
Dependent variable	Youth.Self.Report.Total
Type	OLS linear regression

F(1,1415)	0.0029
R^2	0.0000
Adj. R^2	-0.0007

	Est.	S.E.	t val.	p
(Intercept)	48.3923	0.8391	57.6721	0.0000
imaging	0.0834	1.5599	0.0534	0.9574

Standard errors: OLS

Model Output. This table depicts linear regression model output, including standardized regression coefficients (Est.), standard errors (S.E.), t-values (t val.), and p-values (p-val). Model output also depicts overall model fit statistics and number of observations.

Supplemental References

Achenbach, T. M., Howell, C. T., McConaughy, S. H., & Stanger, C. (1995). Six-year predictors of problems in a national sample: III. Transitions to young adult syndromes. Journal of the American Academy of Child & Adolescent Psychiatry, 34(5), 658–669.

Achenbach, T. M., & Rescorla, L. (2001). Manual for the ASEBA School?-Age Forms & Profiles. Burlington, VT: University of Vermont, Research Center for Children, Youth, and Families.

Avesani, P., McPherson, B., Hayashi, S., Caiafa, C. F., Henschel, R., Garyfallidis, E., Kitchell, L., Bullock, D., Patterson, A., Olivetti, E., & others. (2019). The open diffusion data derivatives, brain data upcycling via integrated publishing of derivatives and reproducible open cloud services. Scientific Data, 6(1), 1–13.

Barratt, W. (2006). The Barratt simplified measure of social status (BSMSS): Measuring SES. Unpublished manuscript. http://socialclassoncampus.blogspot.com/2012/06/barratt-simplified-measure-of-social.html

Bates, D., Mächler, M., Bolker, B., & Walker, S. (2015). Fitting Linear Mixed-Effects Models Using lme4. Journal of Statistical Software, 67(1), 1–48. https://doi.org/10.18637/jss.v067.i01

Dadds, M. R., Maujean, A., & Fraser, J. A. (2003). Parenting and conduct problems in children: Australian data and psychometric properties of the Alabama Parenting Questionnaire. Australian Psychologist, 38(3), 238–241.

Dale, A. M., Fischl, B., & Sereno, M. I. (1999). Cortical surface-based analysis: I. Segmentation and surface reconstruction. NeuroImage. https://doi.org/10.1006/nimg.1998.0395

Dongmei Li (2016). BonEV: An Improved Multiple Testing Procedure for Controlling False Discovery Rates. R package version 1.0. https://CRAN.R-project.org/package=BonEV

Dunn, O. J., & Clark, V. (1971). Comparison of tests of the equality of dependent correlation coefficients. Journal of the American Statistical Association, 66(336), 904–908.

Essau, C. A., Sasagawa, S., & Frick, P. J. (2006). Psychometric properties of the Alabama parenting questionnaire. Journal of Child and Family Studies, 15(5), 595–614.

Fischl, B., Salat, D. H., Busa, E., Albert, M., Dieterich, M., Haselgrove, C., Van Der Kouwe, A., Killiany, R., Kennedy, D., Klaveness, S., & others. (2002). Whole brain segmentation: Automated labeling of neuroanatomical structures in the human brain. Neuron, 33(3), 341–355.

Fischl, B., Salat, D., van der Kouwe, A., & Makris, N. (n.d.). Sé gonne F, Quinn BT, Dale AM. 2004. Sequence-Independent Segmentation of Magnetic Resonance Images. NeuroImage, 23, S69–S84.

Fischl, B., Sereno, M. I., & Dale, A. M. (1999). Cortical surface-based analysis: II: inflation, flattening, and a surface-based coordinate system. Neuroimage, 9(2), 195–207.

Fischl, B., Sereno, M. I., Tootell, R. B. H., & Dale, A. M. (1999). High-resolution intersubject averaging and a coordinate system for the cortical surface. Human Brain Mapping, 8(4), 272–284. https://doi.org/10.1002/(SICI)1097-0193(1999)8:4<272::AID-HBM10>3.0.CO;2-4

Fischl, B., Van Der Kouwe, A., Destrieux, C., Halgren, E., Ségonne, F., Salat, D. H., Busa, E., Seidman, L. J., Goldstein, J., Kennedy, D., & others. (2004). Automatically parcellating the human cerebral cortex. Cerebral Cortex, 14(1), 11–22.

Gaser, C., Dahnke, R., & others. (2016). CAT-a computational anatomy toolbox for the analysis of structural MRI data. Hbm, 2016, 336–348.

Gilmore, A. D., Buser, N. J., & Hanson, J. L. (2021). Variations in structural MRI quality significantly impact commonly used measures of brain anatomy. Brain Informatics, 8(1), 1–15.

Goodman, R. (2001). Psychometric properties of the Strengths and Difficulties Questionnaire. Journal of the American Academy of Child & Adolescent Psychiatry, 40(11), 1337-1345.

Hollingshead, August B. (1957). Two factor index of social position. Unpublished manuscript, Department of Sociology, Yale University, New Haven, Connecticut

Hollingshead, A. B. (1975). Four factor index of social status. Unpublished manuscript, Department of Sociology, Yale University, New Haven, CT.

Johnston, C. A. (2003). Parent and Child Reporting of Negative Life Events: Discrepancy and Agreement across Pediatric Samples. Journal of Pediatric Psychology, 28(8), 579–588. https://doi.org/10.1093/jpepsy/jsg048

Larsen, B., Bourque, J., Moore, T. M., Adebimpe, A., Calkins, M. E., Elliott, M. A., Gur, R. C., Gur, R. E., Moberg, P. J., Roalf, D. R., & others. (2020). Longitudinal development of brain iron is linked to cognition in youth. Journal of Neuroscience, 40(9), 1810–1818.

Liang, J., Shou, Y., Wang, M.-C., Deng, J., & Luo, J. (2021). Alabama Parenting Questionnaire-9: A reliability generalization meta-analysis. Psychological Assessment.

Long, J. A. (2019). interactions: Comprehensive, User-Friendly Toolkit for Probing Interactions. https://cran.r-project.org/package=interactions

Lüdecke et al., (2021). performance: An R Package for Assessment, Comparison and Testing of Statistical Models. Journal of Open Source Software, 6(60), 3139. https://doi.org/10.21105/joss.03139

Revelle, W. (2022). psych: Procedures for Psychological, Psychometric, and Personality Research. Northwestern University. https://CRAN.R-project.org/package=psych

Shelton, K. K., Frick, P. J., & Wootton, J. (1996). Assessment of parenting practices in families of elementary school-age children. Journal of Clinical Child Psychology, 25(3), 317–329.

Thurber, S., & Sheehan, W. (2012). Note on Truncated T Scores in Discrepancy Studies with the Child Behavior Checklist and Youth Self Report.

Wechsler, D. (2014). The Wechsler intelligence scale for children—fifth edition. San Antonio, TX: The Psychological Corporation.

Williams, D. (1971). A test for differences between treatment means when several dose levels are compared with a zero dose control. Biometrics, 103–117.

Wood, S. & Scheipl, F. (2020). gamm4: Generalized Additive Mixed Models using 'mgcv' and 'lme4'. R package version 0.2-6. https://CRAN.R-project.org/package=gamm4
